# Inferring Time‐to‐Speciation From Hybrid Zone Analysis Informs Assessments of Taxonomic Inflation

**DOI:** 10.1111/mec.70361

**Published:** 2026-05-06

**Authors:** Sven Gippner, Katharina Ruthsatz, Christophe Dufresnes, Miguel Vences

**Affiliations:** ^1^ Institute for Cell‐ and Neurobiology Technische Universität Braunschweig Braunschweig Germany; ^2^ Ecology, Evolution and Development Group, Department of Wetland Ecology Doñana Biological Station (CSIC) Sevilla Spain; ^3^ Institut de Systématique, Evolution, Biodiversité (ISYEB), Muséum national d'Histoire naturelle, CNRS Sorbonne Université, EPHE‐PSL, Université des Antilles Paris France

**Keywords:** cline width, hybrid zone, reproductive isolation, species delimitation, taxonomic inflation

## Abstract

Measuring gene flow across hybrid zones can provide a direct evaluation of reproductive isolation (RI) between evolutionary divergent lineages. Geographic‐explicit modelling of gene flow across hybrid zones, known as cline analysis, thus offers a gold standard for species delimitation under the biological species criterion. We here relate divergence time to post‐zygotic RI as inferred from hybrid zone cline width from studies on amphibians, reptiles, birds, mammals and insects. The data confirm an overall significant negative correlation between cline width and divergence time (within‐clade consistently significant only in amphibians). Out of 108 hybrid zones, hybridizing taxa were younger than 10 million years (Ma) in 104 cases, and younger than 7 Ma in 94 cases, implying that older lineage pairs are usually reproductively isolated. In amphibians, the correlation suggested that shallow and steep hybrid zones, characteristic of subspecies and species, are found with divergence times of < 2.6 Ma and > 7 Ma, respectively. In all taxa we revealed a trend of young lineages being increasingly described as new species over time by simulating past sets of taxonomically named species. Overall, the inferred proportion of species with post‐Pliocene ages was highest in birds (27.3%) and mammals (21.8%), but lower in amphibians and squamates (< 10%). In amphibians we document a 56% increase from 1980 to 2010 in the number of newly described species that fall below the hybrid zone‐derived divergence reference point of 2.6 Ma. While this trend so far impacts only a small proportion of the total amount of amphibian species, we commend that taxonomic hypotheses that delimit Pleistocene‐aged lineages as species, or conversely, that consider lineages with Miocene ages or older as conspecific, should be carefully evaluated to avoid taxonomic inflation and deflation.

## 1 Introduction

Species represent a fundamental unit of biological organization (Mayr 1942; de Queiroz 2005) and play pivotal roles in cataloguing, measuring and conserving global biodiversity (Colwell and Coddington 1994; Akçakaya et al. 2000; Coates et al. 2018; Hausdorf 2021). While species were historically perceived as invariable natural entities defined by distinct morphological traits, molecular genetics led to increased precision in detecting variation within them (Ekblom and Galindo 2011; Fujita et al. 2012). As a consequence, challenges to the traditional practice of species detection, diagnosis and description arose (Dufresnes et al. 2023) and numerous species concepts focused on biological properties such as interbreeding, ecological adaptation, diagnosability or monophyly were proposed (de Queiroz 2007; Wilkins 2009). Most of these, however, agree in species being segments of population‐level lineages characterized by discrete evolutionary trajectories, while discordance centers on the operational criteria to recognize such units (Mayden 1997; de Queiroz 1998; Conix et al. 2023).

Lineage divergence is a continuous process and different species criteria thus become traceable at different stages. For instance, the monophyly of populations assigned to a given taxon can arise in very early stages of divergence, and the phylogenetic species criterion (PSC) may thus erroneously delimit groups of individuals that clearly are still conspecific, especially when relying on a small subset of genetic markers. In contrast, the biological species criterion (BSC) is often more conservative because post‐zygotic reproductive isolation (RI) between populations typically arises later along the continuum of divergence (Mayr 1942; de Queiroz 1998, 2007; Sites and Marshall 2004; Padial et al. 2010). According to Agapow et al. (2004), prioritizing the PSC led to 48.7% more recognized species compared to species derived under the BSC. The overreliance on the PSC has been identified as the main factor favouring the recent growth in species numbers in certain vertebrate groups, a tendency named ‘taxonomic inflation’ (Isaac et al. 2004; Zachos et al. 2013) whose causes, impact and prevalence have been intensively discussed (e.g., Agapow and Sluys 2005; Harris and Froufe 2005; Knapp et al. 2005; Zachos 2015; Köhler et al. 2005; Padial and De la Riva 2006; Sangster 2009; Zachos 2015; Tolley et al. 2022).

A cautious approach to avoid and detect taxonomic inflation might be grounded on the BSC and its central indicator, RI, defined as the absence or the fitness costs of hybridization, which influences the exchange rate of genetic material between lineages (Mallet 2005; Mallet et al. 2016). RI can originate at multiple stages of the reproductive cycle (Coyne and Orr 2004); it may be pre‐zygotic, i.e., mediated by behavioural or ecological isolation of the parental species, or post‐zygotic, mediated by hybrid unviability, sterility (caused by genomic incompatibilities) or maladaptation/unattractiveness. For the purpose of this study, we use this BSC‐based approach to define taxonomic inflation as an increase of species numbers driven by assigning species status to lineages showing little or no pre‐zygotic or post‐zygotic reproductive isolation.

Lineage contact is crucial to test for RI in the wild and has thus been integrated in various operational species delimitation procedures (Unmack et al. 2022; Pyron et al. 2023). Especially, zones of secondary contact with the potential of hybridization serve as invaluable ‘natural laboratories’ (Hewitt 1988) to study gene flow and evaluate the extent of RI (Barton 1983; Payseur et al. 2004). Specifically, the transition of allele frequencies at a given locus along a geographic transect that crosses such a hybrid zone (HZ) can be modelled by a sigmoidal cline, whose parameters are affected by the selective (selection against hybrids) and demographic (dispersal) forces at play (Barton and Hewitt 1985, Barton and Gale 1993). Shape and position of clines may vary between loci, and a steep transition of allele frequencies at the same position along the transect, without barriers to dispersal, indicates that post‐zygotic RI stabilized the hybrid zone (Barton and Gale 1993). Multiple factors can mediate geographic patterns of gene flow within HZs (and its heterogeneity across the genome), such as the age since the first contact, historical or ongoing hybrid zone movement, the dispersal behaviour of the taxa involved, and the type and strength of selection experienced by neutral and locally‐adapted alleles when integrated in a foreign environment and genomic background (Hewitt 1988; Buggs 2007; Dufresnes et al. 2020). Nevertheless, HZ analysis is highly useful to quantify reproductive isolation for the purpose of delimiting lineages into species or subspecies (Chambers et al. 2022; Dufresnes et al. 2023; Vences et al. 2024) and can be considered a ‘gold standard’ for testing fulfilment of the BSC. The emergence of high‐throughput sequencing now allows to reliably infer cline width across thousands of genome‐wide, biallelic single nucleotide polymorphisms (e.g., Dufresnes et al. 2021), and an increasing number of case studies are showing the utility of this approach, especially in amphibian taxonomy (e.g., Kalaentzis et al. 2023; Parkin et al. 2023; Purser et al. 2025; Shimada et al. 2025).

The hybrid zone approach to species delimitation is evidently inapplicable to allopatric taxa, i.e., those without secondary contact. Also, it requires dense geographical sampling and substantial molecular resources which clearly are not yet available for many of the thousands of species newly diagnosed and named every year (Miralles et al. 2020). Identifying proxies for RI, namely measurable cues of differentiation shown to covary with successful pre‐ or post‐mating barriers, could help to partly overcome these hurdles. Divergence time is particularly appealing as it can be easily estimated even with single molecular markers (Arbogast et al. 2002). The formation of species is expected to be a clock‐like phenomenon, and the temporal dimension of speciation has accordingly been considered early on (Avise et al. 1998; Hedges et al. 2015). Initial empirical studies based on mitochondrial clocks found pre‐Pleistocene divergence between sister species of North American songbirds, i.e., > 2.5 Ma usually indicating completed speciation (Klicka and Zink 1997; Avise and Walker 1998), and later studies suggested average species ages of typically 2 Ma in birds, 2.2 Ma in mammals, 9.2 Ma in reptiles and amphibians, and 6.8 Ma in fish (Avise et al. 1998). Analysing a comprehensive timetree with over 50,000 species, Hedges et al. (2015) found similar average species ages across vertebrates (2.1 Ma), arthropods (2.2 Ma) and plants (2.7 Ma).

Obviously, these average values involve huge variances (Avise et al. 1998; Hedges et al. 2015) and are inherently biased by the taxonomy and resolution of techniques available at the time. However, other factors may play an even greater role in shaping variation in species formation rates. Reproductively isolated lineages can emerge more rapidly under certain modes of speciation e.g., sympatric settings, hybridization or karyological rearrangements (e.g., Mallet 2007; Abbott et al. 2013; Momigliano et al. 2017; Kautt et al. 2020). Moreover, timetree‐based comparisons may often highly overestimate or underestimate the time needed for species formation, depending on the taxonomic groups. Overestimation occurs in groups where species ages are based on potentially inflated taxonomy, while underestimation is likely in understudied taxa that harbour many undescribed lineages (Linnean shortfall). Based on the observation that, in Palearctic amphibians, intrinsic post‐zygotic isolation (hybrid incompatibilities) accumulates gradually with divergence time, Dufresnes et al. (2021) suggested that it might be possible to predict the species rank following the BSC based on simple estimates of divergence time. By studying hybrid zones, they found that lineages younger than 2 million years tend to exhibit clines wider than 50 km, which given the dispersal capabilities of amphibians, indicate genetic compatibility and thus incomplete species formation. Conversely, hybridizing lineages older than 6 million years exhibited clines narrower than 10 km and a substantial number of loci stopped admixing entirely, which indicates RI and thus an advanced stage of species formation (Dufresnes et al. 2021). A general ‘incompatibility clock’, i.e., an increase of RI with genetic divergence or time is obvious from multiple studies (Mendelson et al. 2004; Coughlan and Matute 2020); for instance, in centrarchid fishes, partial RI was detectable in species pairs with divergences > 6 Ma and pervasive from ca. 15 Ma on (Bolnick and Near 2005). The expectations regarding the capability to hybridize in respect to evolutionary divergence might even serve to test the plausibility of competing biogeographic scenarios when calibrating timetrees (‘lineage‐compatibility plausibility test’; Dufresnes et al. 2024).

For taxonomists who desire to delimit species relying on RI, and thus based on the BSC, such temporal points of reference may therefore serve as yardsticks to evaluate the probability of two lineages to have become species and rank them in the Linnean classification system without the need for cumbersome and sometimes simply impossible hybrid zone surveys. If the relationship between RI and divergence could be comprehensively established across major animal groups, it would strengthen the argument to include divergence time in the toolkit of integrative taxonomists and allow for a more objective testing of taxonomic inflation, i.e., by defining species in an universal manner more proper to comparisons.

Here, we (i) assemble a HZ data set from published studies on a wide range of animals to search for correlations between cline width and divergence time and estimate the time needed for lineages to be considered species in respect to their pattern of introgression. We then (ii) use these time‐to‐speciation estimates to predict taxonomic inflation across the history of taxonomy, based on analysing timetrees of chronologically explicit species assemblages.

## 2 Material and Methods

### 2.1 Compiling a Database of Cline Width Data

We screened the scientific literature as of 14th July, 2022 using the keyword search of Harzing's Publish or Perish version 7.33.338.7819 (Harzing 2007) with the follow keywords: ‘hybrid zone’, ‘cline width’, ‘hzar’ and ‘transect’. Data were considered if the study included information on cline width. Dispersal (McEntee et al. 2020) and divergence time for each pair of hybridizing taxa were compiled prioritizing the most recently published estimates. Dispersal was summarized in eight categories (A: ≤ 0.25, B: > 0.25 and ≤ 0.75, C: > 0.75 and ≤ 2.5, D: > 2.5 and ≤ 7.5, E: > 7.6 and ≤ 25, F: > 26 and ≤ 75, G: > 76 and ≤ 250, H: and > 250 km/generation). A summary of the workflow is given in Figure S1, the full compiled data set is provided in the Dryad repository under https://doi.org/10.5061/dryad.wwpzgmsv2. In general, a HZ could comprise multiple transects, each with multiple cline widths taken at different times. Together with these data, we recorded additional information for each cline analysis, such as the number of markers used to calculate the cline width, the genomic location of markers (mitochondrial or nuclear), and the method used to infer the cline widths (referred to as the ‘inference method’). The inference methods included measurements based on (i) a single marker (referred to as single widths), (ii) median cline widths derived from multilocus cline analyses based on single markers (median widths), (iii) and genomic ancestry or allelic frequency averages (average widths). In cases where only cline widths for multiple single markers were reported, we computed the median width. We divided our data into eight different categories according to this additional information (steps I–III, Figure S1).

HZ cline width reflects the geographic extent of introgression between two lineages and is more reliable when estimated between lineage‐diagnostic loci, i.e., featuring alleles that are fixed between these two lineages (Mallet et al. 1990). Analyses of non‐diagnostic markers, i.e., featuring alleles that are not fixed between species, can bias cline analyses, particularly when geographic sampling does not extend to the parental populations. To identify such markers, we additionally screened for the cline parameters p_min_ and p_max_, which represent the minimum and maximum allele frequencies of the cline (= usually the parental allele frequencies). Consequently, we excluded single and average cline widths obtained from non‐diagnostic clines, as characterized with *p*_min_ > 0.2 and *p*_max_ < 0.8, and we excluded median cline widths when most of the single widths were non‐diagnostic (step IV, Figure S1). Within each of the eight categories of dispersal, we computed the median twice to get one representative cline width, firstly, for each transect (step V, Figure S1) and, secondly, for each pair of hybridizing taxa (step VI, Figure S1).

To quantify the correlation between divergence time and cline width, we first log‐transformed the non‐Gaussian data (Shapiro–Wilk‐Test: < 0.001 in both data series) (Lesaffre et al. 2007). Then, we calculated Pearson correlation coefficients in the resulting log‐transformed dataset (hereinafter, ‘dataset A’)—overall in the entire dataset and in each category respectively—by utilizing the ggpubr package in R version 4.2.1 (Kassambara 2020, R Core Team 2022). In addition, we also calculated the Pearson correlation coefficients for the most complete subset of cline widths in the dataset by selecting those that were inferred by using the ‘average method’ and based on more than one marker. These widths are based on averaged genetic/genomic ancestries without incorporating bias from mitochondrial markers, different inference methods or over‐categorizing (hereinafter, ‘dataset B’).

The effect of divergence time on cline width in dataset A was assessed using a generalized linear model (GLM) in R version 4.2.1 (R Core Team 2022). The potential explanatory variables considered in our models were divergence time, dispersal category and taxonomic group assignment. To avoid bias in cline widths that were calculated by different inference methods, we selected only cline widths from the ‘average widths’ category. To determine the most appropriate probability distribution for the GLM, we compared various distributions using quantile–quantile plots generated with the car package (Fox et al. 2012), resulting in the gamma log distribution as the most suitable probability distribution for our analysis. To identify the best‐fit model among all possible combinations of variables, we employed the glmulti package (Calcagno 2020) using the glm function. This package enabled us to compare and discriminate between different GLMs and select the model that achieved the best balance between goodness of fit and model complexity. We based the model selection process on the weights of the corrected Akaike information criterion (AICc), and the model with lowest AICc was chosen as the preferred model. Finally, an ANOVA‐like test was performed to assess the significance of the best‐model predictors divergence times, dispersal category and taxonomic group on cline width.

### 2.2 Estimating Patristic Distance Change Over Time Periods

We developed an informatic tool that examines changes in the divergence time of taxa over time (https://github.com/iTaxoTools/TaxoInflationAnalyzer). The program is written in Python 3 and requires the DendroPy package (Sukumaran and Holder 2010). It operates on ultrametric trees in Newick format and calculates the patristic distance between species pairs. To analyse specific time intervals, it takes a list of description years (in tsv‐format) as input file and successively excludes taxa based on their description year. By applying this filtering, the mean patristic distances for each defined time interval is then computed. A simple GUI‐driven executable is available from the iTaxoTools project (Vences et al. 2021; https://itaxotools.org).

The output consists of two files for each defined time interval in the dataset: a tree in Newick format comprising all species in the dataset described up to the maximum limit of the set time interval, and a list of species pairs along with their respective patristic distances plus statistical parameters such as sample size, mean patristic distance, and the minimum and maximum patristic distances. Additionally, the program generates a summary file that includes the statistical parameters for all time intervals analysed. This summary file provides a concise overview of the patterns observed throughout the entire analysis. A second program mode compiles, for a user‐defined patristic distance threshold, (i) the number of species pairs falling below the defined threshold and (ii) their corresponding proportion.

As input, we generated ultrametric trees using the timetree.org website in June 2022 (Kumar et al. 2022). To ensure the validity of tips, we developed a workflow to filter out invalid names and obtain a list of species with their corresponding description years. Firstly, a timetree for each taxonomic group was generated using the ‘Build a Timetree’ function provided on the timetree website (https://www.timetree.org). The taxon names were then extracted from the generated timetree using the R package *ape* (Paradis and Schliep 2019) and non‐binomial names were excluded. The extracted list was cross‐checked with the GBIF Backbone Taxonomy (GBIF Secretariat 2021) using the ‘species matching tool’ (https://www.gbif.org/tools/species‐lookup, accessed June 2022). Matches at the species rank with an accuracy of 100% were extracted while matches with lower accuracy were manually checked. The resulting species list, along with their respective description years, was re‐uploaded onto timetree.org using the ‘Load a List of Species’ function. The newly generated timetrees were then downloaded and used as input files.

### 2.3 Temporal Calibrations for the Analysis of Divergence Times

For Lissamphibia our HZ analysis suggested well‐defined empirical values for time‐to‐speciation derived from the correlation analysis between divergence time and cline width (see Results). To calibrate the analysis for Lissamphibia, we utilized the cline transition classification proposed by Dufresnes et al. (2021) with three categories: shallow (> 50 km), steep (between 50 km and 10 km) and very steep (< 10 km). We estimated points of reference for time‐to‐speciation with the stat_ellipse function from the R package ggplot2 to calculate the multivariate *t*‐distributions for average cline widths categorized as shallow and very steep (> 2 markers), respectively (Wickham et al. 2022). The maximum *t*‐value from the shallow HZs served as the first point of reference, indicating a point beyond which we expect reproductive isolation to be mostly completed. Conversely, the minimum *t*‐value from the very steep distribution represented the second point of reference, below which we assume reproductive isolation to be incomplete in most species.

We then applied the same workflow to seven taxonomic groups: birds (Aves), squamates (Squamata), butterflies (Lepidoptera), molluscs (Mollusca), mammals (Mammalia) and neoteleost ray‐finned fishes (Neoteleostei), but using a more conservative reference point for time‐to‐speciation (2 Ma) to detect very young (post‐Pliocene) species pairs. In addition, mean divergence times of all species pairs was compared between animal groups by using a Bonferroni calibrated pairwise *t*‐test (*rstatix* package) in R (Kassambara 2021).

## 3 Results

### 3.1 Dataset Description and GLM Results

A total of 2078 cline widths were obtained through literature screening, representing 259 sampled transects and 160 distinct hybridizing pairs of lineages. Cline width data, as well as data on divergence times and dispersal rates, were compiled from published studies across twelve organismal groups (for a complete list of all sources see Table S1). After applying our filtering workflow (Figure S1), and excluding HZs without divergence time data, dataset A consisted of 232 categorized cline widths for 108 pairs of hybridizing lineages (Table 1; metadata and original references available under https://doi.org/10.5061/dryad.wwpzgmsv2). Within dataset A, 81 cline widths were estimated using the average method, 57 using the median method, and 94 were based on single markers (77 mitochondrial), measured across 108 hybridizing pairs of lineages. Dataset B is a filtered subset of dataset A and includes 71 of its cline widths, all estimated using the average method. This final dataset includes only one cline width per hybridizing pair of lineages across five organismal groups, representing 65.7% of all taxon pairs and 30.6% of the cline widths in dataset A (Table 1).

**TABLE 1:** Overview of hybridizing pairs of lineages and their cline widths included in dataset A and B, summarized by major organismal group.

Organism group	Pairs of hybridizing lineages (and number of cline widths) in dataset A	Pairs of hybridizing lineages in dataset B	Proportion of pairs (and cline widths) from A present in B
Aves	28 (61)	19	67.9% (31.1%)
Hexapoda	13 (24)	5	38.5% (20.8%)
Lissamphibia	32 (69)	25	78.1% (36.2%)
Mammalia	12 (34)	8	66.7% (23.6%)
Squamata	18 (34)	14	77.8% (41.2%)
Other	5 (10)	0	0% (0%)
Total	108 (232)	71	65.7% (30.6%)

*Note:* Dataset B represents a filtered subset of dataset A retaining one multi‐marker cline width per hybridizing taxon pair. Primary sources for all hybrid zone data included in datasets A and B are provided in the Dryad repository; a complete list of all screened studies (*n* = 203) is also available there. The organism group ‘other’ comprises Actinopterygii, Crustacea, Mollusca, Testudinata.

In dataset A, the range of cline widths spanned over five orders of magnitude, from 0.02 km in two European shrew species *Sorex araneus* / *S. antinorii* up to 1065.4 km in a HZ formed by two lineages of the European barred grass snake (*Natrix helvetica*). The divergence times ranged from 0.014 Ma in two lineages of the European common vole ( *Microtus arvalis* ) up to 21.5 Ma in the North American salamanders (*Ensatina e. eschscholtzii/klauberi* and *E. eschscholtzii platensis/xanthoptica*). The divergence times (mined from the literature) of 12 pairs of lineages were older than 7 Ma in the dataset A (five older than 10 Ma). Among these very old lineages, gene flow was detected but was locally restricted in the European fire‐bellied toads (*Bombina bombina/variegata*, 7.7 Ma), the European partridges ( *Alectoris rufa* /*graeca*, 8.5 Ma), the Iberian lizard species (*Lacerta lepida/nevadensis*, 9.0 Ma, now *Timon lepidus*/*nevadensis*), two lineages of the Japanese salamander ( *Cynops pyrrhogaster* , 9.7 Ma), North American newts (*Taricha t. torosa/sierrae*, 10.0 Ma, now regarded as distinct species), European grass snake species (*Natrix natrix/helvetica*, 7.8 Ma and *N. astreptophora*/*helvetica*, 10.6 Ma), two Caribbean anole subspecies (*Anolis d. distichus/ignigularis*, 11.0 Ma), and the North American salamanders (*E. e. eschscholtzii/klauberi* and *E. eschscholtzii platensis/xanthoptica*, 21.5 Ma). Dataset A also included comparably young lineages with hybrid zone patterns suggesting distinct species. These were predominantly found in birds and mammals, e.g., South American antbirds (*Hypocnemis ochrogyna/striata*, 1.5 Ma), sex‐role reversed Neotropical jacanas (*Jacana jacana/spinosa*, 0.7 Ma), or the European shrews ( *Sorex araneus* / *S. antinorii* , 0.3 Ma).

The model selection process yielded 26 different GLMs (Figure S2). The best‐fit GLM for predicting cline width was AICc weighted with 88.7% (Table S2) and incorporated three variables: divergence time, dispersal rate category and taxonomic group. While all three explanatory variables were included in most of the high‐weighted models, interactions between these variables were only present in a minority of the tested models (Figure S3). The ANOVA results indicate that all predictors—divergence times, dispersal category and taxonomic group—significantly influenced mean cline width (*p* < 0.01, Table S3). Among these, dispersal category and divergence times were found to be highly significant (*p* < 0.001, Table S4). Detailed effects of specific categories within these predictors are further outlined in Table S5.

### 3.2 Correlation Analysis Between Divergence Time and Cline Width

**FIGURE 1:**
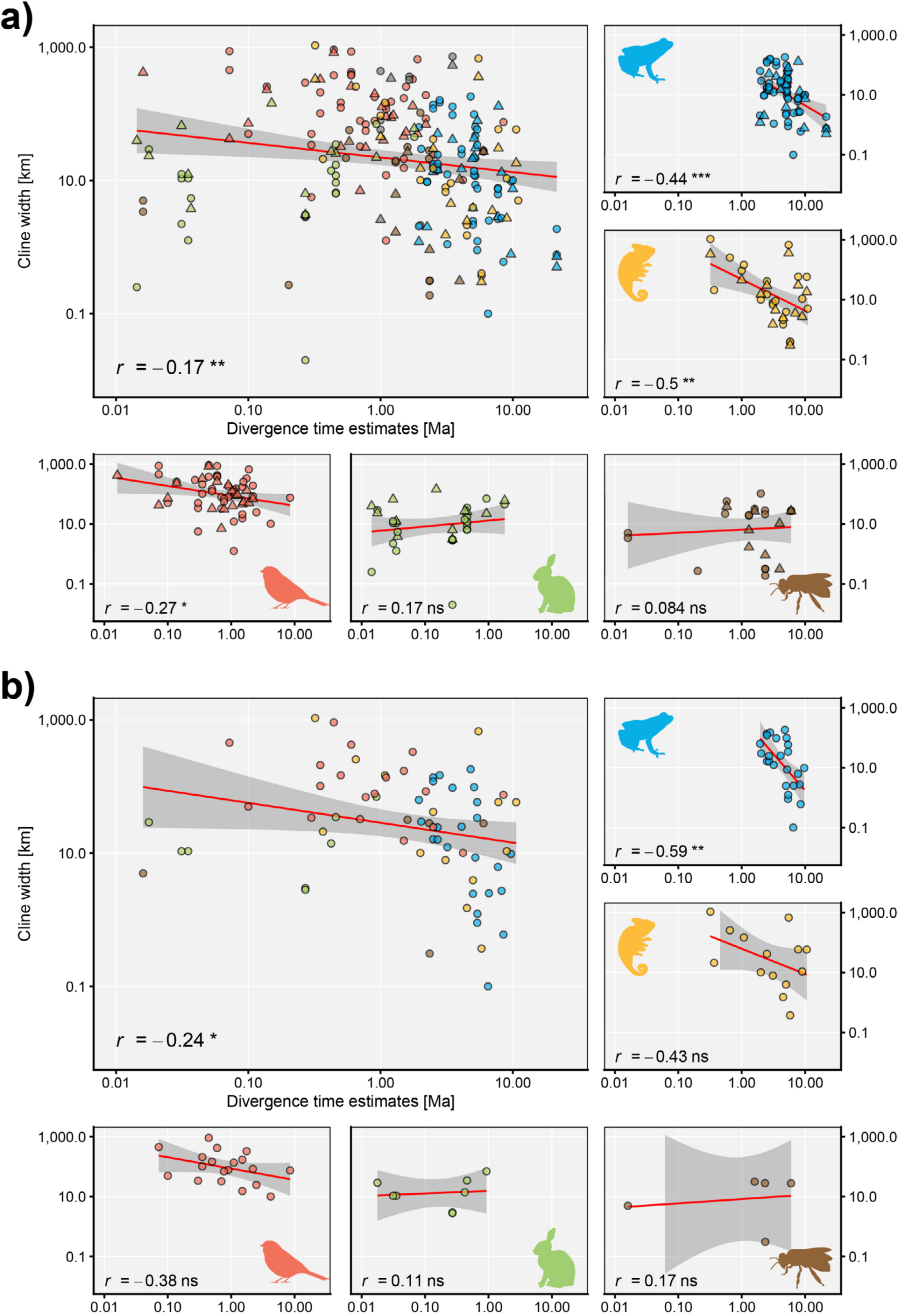
Linear relationship between log‐transformed published cline widths and divergence time estimates in two differently filtered datasets divided into five hybridizing organism groups. Upper plot (a) shows the dataset A comprising 232 cline widths (averaged based on eight different categories) of 108 hybridizing taxa with. Lower plot (b) shows the dataset B (only cline widths based on more than one marker and measured by using the average method) comprising 71 hybridizing taxa. The respective upper left graphs in both (a) and (b) show hybrid zones of all higher taxa combined and includes cline widths of five hybrid pairs from other taxonomic groups (grey) for dataset A. Cline widths derived from mitochondrial markers are shown in a triangle shape. For each plot the Pearson correlation coefficient (*r*) and its *p*‐value (ns, *p* > 0.05; *p < 0.05; **p < 0.01; *** *p* < 0.001) was calculated from a linear regression (red line, 95% confidence interval indicated by surrounding grey area). Axes are log scaled.

A significant overall negative correlation between log‐transformed cline width and divergence time estimates was found in both dataset A (108 hybridizing taxa based on 232 cline widths, *R* = −0.17, *p* = 0.008, Figure 1a) and B (*n* = 71, *R* = −0.24, *p* = 0.045, Figure 1b). Notably, the most consistent correlation was detected in amphibians (dataset A: 32 hybridizing taxa based on 69 cline widths, *R* = −0.44, *p* = < 0.001, Figure 1a; representative dataset: *n* = 25, *R* = −0.59, *p* = 0.002, Figure 1b). In squamates and birds, significant negative correlations were found in dataset A but did not persist in dataset B, although trends were still present (Figure 1a,b). In mammals and hexapods, the groups with smallest sample size, a counterintuitive positive trend was observed, but no significance was detected, and the *R* values were comparatively the closest to zero (Figure 1a,b).

Within dataset A (108 pairs of hybridizing lineages), a negative trend between divergence time estimates and cline width was observed in taxonomic groups with larger sample sizes (Lissamphibia, Aves, Squamata) and more often when cline widths were measured based on multiple markers using the average method (Figures S4a–S9a; significant in amphibians HZs based on more than 100 markers). Expectedly, trends of inverse (positive) correlations were found between divergence time and selection coefficient (cline widths corrected by discrete dispersal rates, Figures S4b–S9b; inconsistent in data sets with smaller sample sizes).

### 3.3 Ages of Amphibian Species According to Time of Description

The divergence time of hybridizing lineages obtained independently from either direct literature mining or the timetree.org database were significantly correlated (*R* = 0.57, *p* < 0.001, Figure S10). For amphibian hybrid pairs (*n* = 14) divergence times in timetree.org were older (mean = 8.63 Ma, median = 7.9 Ma, standard deviation (SD) = 4.64 Ma) compared to the divergence times we extracted directly from the literature which included population genetic rather than phylogenetic estimates (mean = 5.3 Ma, median = 5.38 Ma, SD = 1.96 Ma). Based on the timetree‐derived divergence times, amphibian species pairs with one or both species described in the most recent decade of our analysis (2000–2010) had a median divergence time of 9.12 Ma (*n* = 399, minimum divergence time = 0, max. div. *t*. = 81.1 Ma, SD = 9.96 Ma).

**FIGURE 2:**
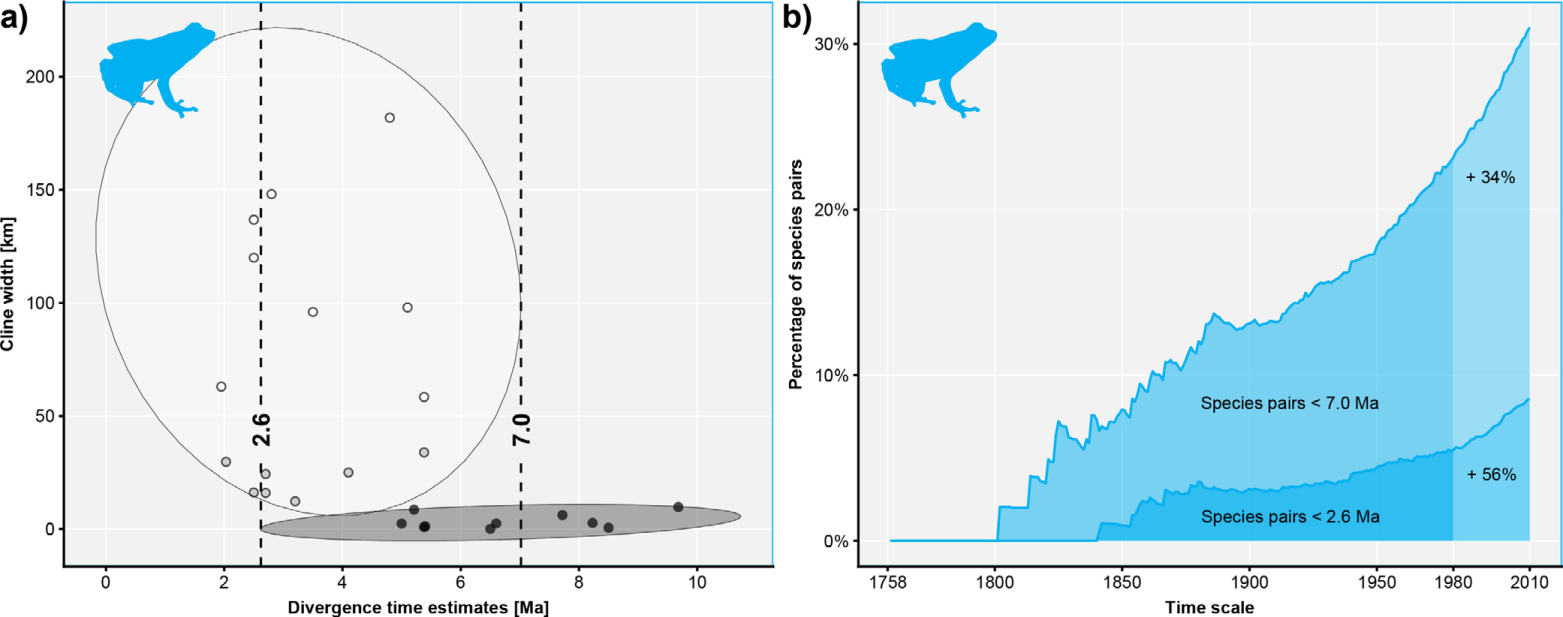
Hybrid zone‐derived points of reference for time‐to‐speciation, used to detect taxonomic inflation in Lissamphibia. (a) Amphibian hybrid zones classified as shallow (cline width above 50 km, white dots), steep (cline width between 10 and 50 km, grey dots), and very steep (cline width under 10 km, black dots). Estimation of presumably completed (> 7.0 million years, Ma) and presumably not completed (< 2.6 Ma) reproductive isolation in amphibian hybrid zones by aligning tangents to a multivariate *t*‐distribution of shallow and very steep hybrid zones, respectively. (b) Percentage of Lissamphibia species pairs with time‐to‐speciation estimates below the points of reference of 2.6 Ma and 7.0 Ma plotted on a time scale starting with the beginning of the Linnean Nomenclature in 1758. The percentages were obtained based on data from an amphibian timetree (*n* = 2523 taxa) downloaded from timetree.org and aligned with GBIF backbone taxonomy.

We estimated two reference points for time‐to‐speciation in amphibians using (i) the tangent of the *t*‐distribution at the x‐maximum of ‘shallow’ (7.0 Ma, derived from eight hybridizing taxa) and (ii) the x‐minimum of ‘very steep’ cline widths (2.6 Ma, derived from ten hybridizing taxa, Figure 2a). When compared with timetree‐derived divergence times, these benchmarks revealed 216 species pairs (8.6%) younger than 2.6 Ma and 782 species pairs (31%) younger than 7 Ma in the amphibian timetree (*n* = 2523 taxa). Over the decades of taxonomic history, the percentage of species pairs falling under 7 Ma steadily increased, reaching 17.8% in 1950 (Figure 2b) whereas the species pairs younger than 2.6 Ma remained at 3%–4% between 1867 and 1950. Since 1950, both proportions continuously increased. Growths of 34% for species pairs younger than 7 Ma and 56% for species pairs younger than 2.6 Ma were noted between 1980 and 2010 (Figure 2b).

### 3.4 Ages of Species Over Time in Other Animal Taxa

**FIGURE 3:**
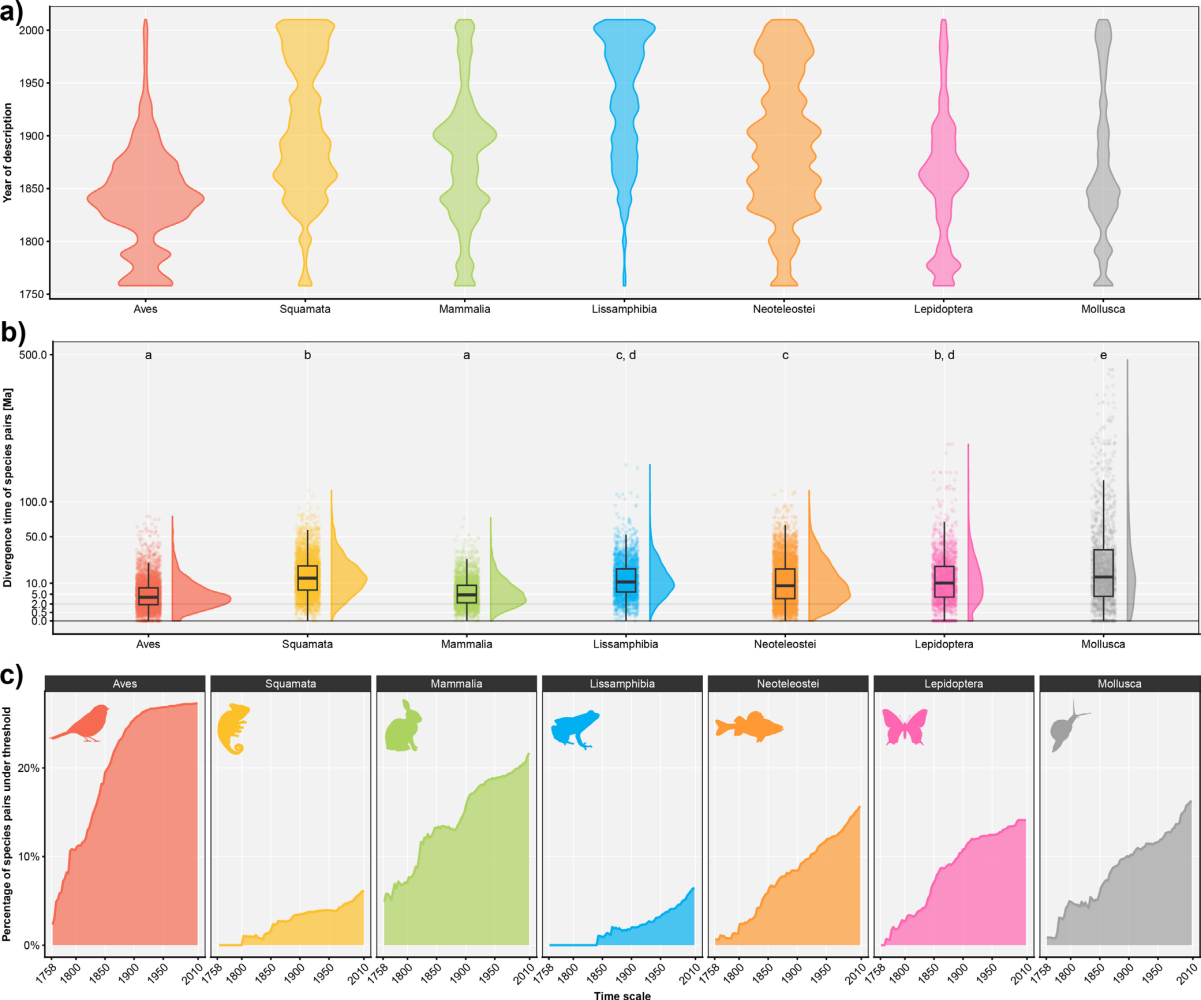
Ages (divergence times from sister taxon) of species in seven taxonomic groups. Based on trees from timetree.org and on species names aligned with the GBIF backbone taxonomy. (a) Number of species descriptions over years (1758–2010). (b) Distribution of divergence times between species pairs in million years (Ma). Shared lower case letters denote non‐significant differences in means (Bonferroni adjusted *t*‐test). (c) Proportion of species pairs younger than 2 Ma.

Our data reveal substantial differences across higher taxa both in species description rates from 1758 to 2010 (Figure 3a), and divergence times of species pairs known at the respective periods of taxonomic history (Figure 3b; significant for all comparisons except Aves vs. Mammalia, Lissamphibia vs. Neoteleostei, Squamata vs. Lepidoptera and Lissamphibia vs. Lepidoptera). The Mollusca timetree displays the highest mean, median and standard deviation for all species pair divergence times (mean = 35.5 Ma, median = 13.6 Ma, SD = 60.8 Ma, Table S5) whereas the bird timetree has the lowest median with 3.9 Ma. The percentage of species pairs < 2 Ma also varies among taxonomic groups (Figure 3c, Table 2), with the highest percentage inferred for birds and mammals with 27.3% and 21.8%, respectively (Table 2), and the lowest percentage in squamates and amphibians with 6.1% and 6.6% (Table 2). The highest proportions of species pairs < 2 Ma and having one or both species described between 1980 and 2010 were also found in mammals and birds (48.1%/45.1%, Table 2), while the lowest proportions were observed in amphibians and squamates (11.8%/11.7%, Table 2). An increase in recent descriptions of species < 2 Ma was evident in all taxonomic groups (Table 2).

**TABLE 2:** Increases of descriptions of species with post‐Pliocene divergences to their closest relative in the period 1980–2010.

Timetree	SP	SP < 2 Ma [%]	SP (1980–2010)	SP < 2 Ma (1980–2010) [%]	Increase by (%)
Mammalia	2887	629 (21.79%)	237	114 [48.10%]	120.78
Neoteleostei	5419	860 (15.87%)	659	230 [34.90%]	119.92
Mollusca	1565	258 (16.49%)	201	71 [35.32%]	114.27
Squamata	3989	245 (6.14%)	804	94 [11.69%]	90.36
Lepidoptera	2109	296 (14.04%)	106	28 [26.42%]	88.21
Lissamphibia	2523	168 (6.66%)	769	91 [11.83%]	77.71
Aves	4415	1206 (27.32%)	51	23 [45.10%]	65.10

*Note:* Species pair proportions of recently described species (1980–2010) compared to the overall proportion of species pairs younger than 2 million years (Ma). Timetrees were generated with timetree.org. SP—total species pairs, SP < 2 Ma—species pairs younger than 2 million years [and in percent], SP (1980–2010)—species pairs with one species described between 1980 and 2010, SP < 2 Ma (1980–2010)—number of species pairs younger than 2 Ma with one species described between 1980 and 2010.

## 4 Discussion

### 4.1 Hybrid Zone Cline Width Scales With Divergence Time

In this study, we contribute an extended perspective on the relationship between the divergence time of hybridizing lineages and the width of the hybrid zones they form in the wild, by examining 108 pairs of lineages from different clades including Squamata, Aves, Mammalia, Hexapoda and Lissamphibia. This considerably widens the scope of similar studies that remained limited to a given taxonomic group, e.g., on Australian squamates (Singhal and Moritz 2013; Singhal and Bi 2017) or Palearctic anurans (Dufresnes et al. 2019, 2021). For the combined data set of all higher taxa, we found a significant correlation between divergence time and HZ width and identified divergence time as the most important predictor of HZ width in the preferred GLMs. The correlation was significant in amphibians, squamates and birds but not in mammals or hexapods, with the clearest and most consistent signal observed in amphibians. In this group, the relationship between divergence time and hybridizability was hypothesized to reflect the gradual progress of post‐zygotic isolation with genetic divergence via the random accumulation of multiple genetic incompatibilities across the entire genome, each with a small effect on hybrid fitness (the ‘mass of genes’ model for the progression of post‐zygotic isolation, Dufresnes et al. 2021). Importantly, in our survey we only found very few examples of hybrid zones between pairs of lineages older than 10 Ma, indicating that after this time span, species formation is completed in the vast majority of lineages. Our results suggest that this model might also apply to other vertebrate groups, and that the time of divergence is accordingly the key determinant of speciation, at least under allopatric processes (Dufresnes et al. 2021).

One of the implications of this model is that genetic incompatibilities eventually become expressed with a ‘snowball effect’ (Matute et al. 2010; Moyle and Nakazato 2010). In other words, once a certain number of mutations has arisen between diverging lineages, introgression substantially depletes hybrid fitness by breaking epistatic functions, i.e., which rely on co‐evolving genes. Variation in the pace of this mechanism is expected given the differences in substitution rates among organisms (Brownstein et al. 2024). In taxa with slow mutation rates and slow substitution rates caused by factors such as long generation time, slow metabolic rate or low fecundity (e.g., Martin and Palumbi 1993; Bromham 2020; Bergeron et al. 2023), longer periods might be needed to reach the number of random barrier‐loci required for RI to arise. This might, for instance, explain the particularity of the evolutionary static ‘living fossils’ to retain the ability to hybridize despite many millions of years of independent evolution (Brownstein et al. 2024). The random accumulation of incompatibilities, together with fluctuations in gene flow in the hybrid zones due to demographic processes, also imply that, stochastically, not all loci are affected in the same way, and studies analysing a small number of loci incur the risk of detecting idiosyncratic signals of those markers that may not accurately correlate with RI; however, the signal of hundreds or thousands of loci in genome‐scale datasets will usually correlate well with average genomic ancestry.

For insects and mammals, the absence of a significant pattern in our dataset may be explained by relatively limited sample sizes for these taxa, but may also reflect uncertainties in divergence time inference or intra‐clade variation in substitution rates. Additionally, differences in the underlying mechanisms of speciation mechanisms may contribute to this pattern. For example, fishes, here represented by only four hybrid zones grouped as ‘other’ in dataset A, may more frequently undergo sympatric speciation and adaptive radiation. In these cases, pre‐zygotic reproductive isolation, through local adaptation and sexual selection, might act as the initial driver of divergence rather than prolonged allopatry (Kautt et al. 2020; Ngoepe et al. 2023), perhaps because their diverse aquatic environments facilitate niche differentiation even within the same geographic area. The HZ approach to species delimitation thus relies on the assumption that species formation is primarily driven by a gradual—largely linear—accumulation of genomic divergence, and may not be applicable in systems mainly driven by intrinsic and extrinsic processes involving pre zygotic isolation by fast behavioural, ecological or morphological divergence as they are regularly reported for insects and fishes (e.g., Endler 1977; Xue et al. 2009; Henrich and Kalbe 2016). In general, it is obvious that many confounding factors influence any cross‐taxa analysis of hybrid zones, yet our analysis suggests that this background noise is still insufficient to hide the important role that progressive genome‐wide divergence plays in species formation across different animal groups.

### 4.2 Hybrid Zone Data Estimate Points of Reference for Time‐to‐Speciation in Amphibians

Due to differences in the presence and strength of a correlation between HZ width and divergence times among the major taxa in our analyses, we limited the estimation of benchmark values for time‐to‐speciation to amphibians, where the correlation was the clearest (see also Dufresnes et al. 2021). We expected major differences in correlation pattern due to vastly different dispersal abilities of the taxa involved (McEntee et al. 2020), a confounding factor that may even substantially vary between closely related species (Stevens et al. 2010). We attempted to account for this factor by using the coefficient of selection against hybrids, an ad hoc way to correct HZ width by dispersal and thus only quantify the effect of RI on the width. However, we did not retrieve stronger correlations by using this coefficient. A reason for this might be that quantifying dispersal in itself is methodologically challenging and error‐prone, as it requires indirect estimation methods such as mark‐recapture or genetic inferences and the mathematical models that rely upon these methods (Nathan 2001). Moreover, the literature on animal movement is primarily focused on migration within home ranges rather than dispersal, and these estimates, although informative of the relative mobility of species, might be spurious to predict how fast genes diffuse in the geographic space generations after generations (Barton and Hewitt 1985; Araya‐Donoso et al. 2022).

A potentially more important confounding factor that we could not control for, is the uncertainty of divergence time estimates, all collated (by ourselves and timetree.org) from a methodologically heterogeneous array of primary publications. As an example for extreme variation of these estimates, the median divergence time of the European toads *Bombina bombina* / *B. variegata* ranges from 5.9 Ma (Fromhage et al. 2004) to 60 Ma (Igawa et al. 2008). However, it is advised, when feasible, to delimit species at a ‘local’ taxonomic scale, namely based on divergence points of reference calibrated for a given group of species and a given methodology. This will help to mitigate the variation in time estimates and dispersal capabilities, thereby improving the reliability and thus predictive potential of divergence‐HZ width correlations (Dufresnes et al. 2021, 2023).

Despite these uncertainties, the correlation between HZ width and divergence time in amphibians was remarkably strong in our analysis and allowed us to estimate, for this taxonomic group, two statistically derived divergence points of reference that we consider to be indicative of weak or absent post‐zygotic RI (< 2.6 Ma), or conversely, indicative of presence of substantial post‐zygotic RI (> 7 Ma). These estimates obviously must not be seen as dogmatic ‘threshold’ criteria of species delimitation, given the expected variation in the time‐to‐speciation discussed above, but should rather be interpreted as one of several elements to consider when delimiting species under integrative taxonomy. That said, we recommend that taxonomic hypotheses separating < 2.6 Ma amphibian lineages as distinct species, or considering > 7 Ma lineages as conspecific, should be reviewed in depth by such integrative analyses. Moreover, noting that anuran hybrid zones wider and narrower than 30 km are typically associated with few or many barrier loci, and thus respectively characterize subspecies and species (Dufresnes et al. 2021), the ‘half‐time’ to speciation i.e., the divergence time corresponding to a cline width of 30 km in the amphibian correlation, is 4.1 My (CI: 3.5–4.8 My), which can be also informative for evaluating species delimitation hypotheses in what has been called the grey zone of speciation (Dufresnes et al. 2021; Roux et al. 2016). Although the relationships in other taxa are less strong from the available data, we similarly found only very few HZs older than 10 Ma (none older than 22 Ma), and HZs of lineages > 7 Ma always were characterized by limited gene flow. In fact, the significant correlations in squamate and birds imply that, as for amphibians, we can compute the ‘half‐time’ to speciation from the regression formulas. Again using 30 km clines as the inflexion point, the corresponding divergence times would be 2.6 My (CI: 1.9–3.6) for squamates and 0.9 My (CI: 0.6–1.3) for birds. These values are here only given to exemplify the approach, and should be revised based on stronger divergence‐cline width correlations (e.g., computed from more hybrid zones), and most importantly, by establishing the actual average cline width separating species and subspecies in these groups. For instance, it is likely that hybrid zones much wider than 30 km of cline width still characterize valid species in birds, which usually have a much higher dispersal potential than amphibians. Future studies should thus determine the average cline widths that associate with reproductive isolation in groups with different dispersal capabilities to then establish which divergence times correspond to these values.

The timeframes of speciation in different groups suggest that conspecific lineages older than 7 My are rare across animals, despite the possibility of occasional hybridization especially in living fossil lineages with low substitution rates (Brownstein et al. 2024). Conversely, the dataset includes comparably young lineages with species‐level divergence predominantly in mammals and birds, consistent with the findings of Avise et al. (1998). This trend is also evident in timetree.org, where the average patristic distance of species‐pairs in mammals and birds is significantly lower than in other analysed groups (Figure 3b). The shorter time to speciation of mammals relative to amphibians is well‐known since the early studies of Wilson et al. (1974) who related it to a higher ratio of regulatory evolution to protein evolution. The underlying mechanisms may lead more swiftly to patterns of gene misexpression in hybrids (Prager and Wilson 1975), but the underlying mechanisms of these patterns are still poorly understood (Runemark et al. 2024). Prager and Wilson (1975) also found that the potential for interspecific hybridization is lost faster in mammals than in birds (see also Fitzpatrick 2004); however, fast speciation in birds may be caused by pre‐zygotic RI, where colour pattern or song differences lead to completed species formation, despite a lack of full post‐zygotic RI (Edwards et al. 2005).

### 4.3 Defining and Detecting Taxonomic Inflation Is Challenging

Our analysis suggests that the number of newly described amphibian species below the HZ‐defined point of reference for time‐to‐speciation increases during the history of taxonomy. Interpreting this pattern as a sign of taxonomic inflation first requires a precise definition of this term. Taxonomic inflation initially described what some authors perceived as an unnecessary oversplitting of taxonomic ranks (Patterson 1999; Alroy 2003). It was subsequently used to address an increase of numbers in the species rank, particularly by elevating subspecies to species (Isaac et al. 2004). Padial and De la Riva (2006) then distinguished between ‘taxonomic inflation *sensu stricto*’, i.e., the growth of species numbers due to the elevation of subspecies, and ‘taxonomic inflation *sensu lato*’, the growth in species numbers in certain genera resulting from the adoption of lineage‐based criteria in species delimitation.

Here, we propose a refined definition of taxonomic inflation as an increase of species numbers driven by the acceptance of lineages that show no or weak signs of pre‐zygotic or post‐zygotic reproductive isolation as species. More precisely: taxonomic inflation in our definition consists of adding presumed species to the Linnean classification that under (potential) co‐occurrence lack behavioural, ecological, or genetic barriers to reproduction and would produce freely admixing populations without selective pressures against hybrids and eventually result in lineage fusion. Applying time‐to‐speciation yardsticks—such as 2.6 Ma in amphibians—to lineages where reproductive isolation is inherently difficult to detect (i.e., allopatric taxa) offers a rough guideline for identifying potential cases of taxonomic inflation, which can then be subjected to closer taxonomic examination. The calculation of mean patristic distances in ultrametric trees by progressively excluding taxa based on their year of description, as used herein, facilitates the detection of signals of taxonomic inflation. Of course, any particular time estimate from such timetrees is associated to substantial uncertainty (Graur and Martin 2004). Our analysis may also be influenced by regional biases affecting the species included in HZ studies (McEntee et al. 2020) and those for which timetree data are available, for instance by overrepresentation of species from North America and Europe, i.e., at latitudes where speciation is suspected to be slower (Weir and Schluter 2007). Also, a time lag for divergence date information of new species becoming available in the timetree.org database may distort the numbers of species described per year represented in the analysis compared to overall numbers. Finally, our approach only focuses on new species descriptions and does not detect upgrades of previously described subspecies, which possibly explains relatively high description rates of poorly divergent bird and mammal species early on in taxonomic history: in these taxa, naming subspecies has a long tradition, and after subsequent elevation to species, they will be counted for the year of original description, potentially decades earlier than their actual recognition at the species level. For amphibians this issue is of less relevance, given that only 14.3% species newly recognized between 1992 and 2003 can be attributed to the elevation of subspecies to species level or revalidations of synonyms (Köhler et al. 2005).

### 4.4 Evolutionary Ages of Newly Described Species in Amphibians Decrease Over Time

As taxonomists progressively add species to the catalogue of life on our planet, it is to be expected that the inferred evolutionary ages of newly described species will on average decrease. This is on one hand due to purely stochastic reasons, because the more species the catalogue contains, the higher the probability that this also includes a close relative of a newly described species. On the other hand, it is obvious that taxonomists and collectors will first recognize those species as new that are highly distinctive in morphology, which implies less distinct species—often separated by small evolutionary ages from their close relatives—will be described at a later stage of taxonomic history. Finally, methodological innovations over time increasingly allow recognizing species that are young and morphologically and genetically very similar, but still a biological reality. None of these factors constitutes taxonomic inflation itself, but all lead to the same empirical pattern: more recently described species are morphologically more similar and exhibit lower evolutionary divergences from, already described species.

Conforming to this expectation, we found increasing proportions of young species for all higher taxa examined. The proportion of post‐Pliocene species (< 2 Ma) newly described also rises in all groups, and from 1980 to 2010 increased by 48.1% and 45.1%, respectively, in mammals and birds (Table 2). These two groups are, however, known to contain a large number of young species, and it is therefore more relevant to examine proportions of particularly young taxa in amphibians where HZ data were sufficiently consistent to infer points of reference for time‐to‐speciation. In amphibians we revealed a 56% increase in the number of newly described species younger than 2.6 Ma from 1980 to 2010, although the overall proportion of species < 2.6 Ma remains very low (< 10% until 2010; Figure 2). Obviously, many of the newly described young species of amphibians are backed up by convincing integrative evidence, and some of them may even be thoroughly supported by in‐depth population genomic data. However, in other cases, newly described species might be more controversial due to incomplete sampling of geographical variation, weak morphological differences and reliance on single molecular markers. Moreover, some species‐delimitation programs, originally designed to work with a few genetic markers, may misinterpret population‐level divergence as species‐level divergence when applied to the thousands or hundreds of thousands of SNPs generated by high‐throughput sequencing (Sukumaran and Knowles 2017; Leaché et al. 2019, 2021; Chambers and Hillis 2019; Dufresnes et al. 2020; Burriel‐Carranza et al. 2023). In cases where RI is unlikely and species status cannot be confirmed, a subspecies classification might be worth considering (Hillis 2020; de Queiroz 2020; Dufresnes et al. 2023).

Species are fundamental units for important fields of science and society which will be substantially affected by taxonomic inflation and more generally, by the instability of species lists (Frankham et al. 2012). Taxonomists race against time to discover and describe the millions of scientifically unnamed new species on Earth (de Carvalho et al. 2007; Costello et al. 2013; Murali et al. 2023). Describing poorly delimited lineages as species diverts much‐needed work capacity to re‐assessing their taxonomy (e.g., Tolley et al. 2022), and away from the pressing task to chart our planet's uncatalogued diversity. The results obtained from timetree‐derived patristic distances of species pairs provide a means to quantify the rise of suspiciously young species described over time, but additional work is needed to attribute any detected trend to taxonomic inflation. In mammals and birds, where the increase in the proportion of post‐Pliocene (< 2 Ma) species described between 1980 and 2010 was particularly marked, more in‐depth studies of hybrid zones are necessary to understand if points of reference for time‐to speciation can be established for subgroups of homogeneous life and natural histories. In amphibians, it will be a worthwhile endeavour to re‐examine species pairs < 2.6 Ma to understand how many of these are convincingly well‐differentiated species (perhaps even sympatrically occurring, or with different advertisement calls: Vences et al. 2012) rather than potentially intraspecific lineages described without sufficient evidence.

## Author Contributions

Conceptualisation: Sven Gippner, Christophe Dufresnes and Miguel Vences. Methodology: Sven Gippner and Christophe Dufresnes. Investigation: Sven Gippner and Christophe Dufresnes. Formal analysis and validation: Sven Gippner, Christophe Dufresnes and Katharina Ruthsatz. Writing – original draft: Sven Gippner, Christophe Dufresnes and Miguel Vences. Writing – review and editing: Sven Gippner, Katharina Ruthsatz, Christophe Dufresnes and Miguel Vences.

## Funding

M.V., C.D. and S.G. were supported by a grant of the Deutsche Forschungsgemeinschaft (VE247/19‐1) in the framework of the Taxon‐Omics priority program (SPP1991).

## Conflicts of Interest

The authors declare no conflicts of interest.

## Supporting information


**Figure S1:** The workflow for processing cline widths from hybrid zone literature and incorporating them into the database used in this study.
**Figure S2:** Information criteria (IC) values for all tested models. A red horizontal line denotes the 2 IC units' threshold.
**Figure S3:** Relative importance of the generalized linear model terms across all models. D, dispersal rate category; G, taxonomic group; T, divergence time.
**Figure S4:** (a) Mean cline widths in kilometres (km) and (b) dispersal‐corrected selection coefficient values plotted against the estimated divergence time of the involved taxa in million years (Ma) for all 109 published hybrid zones. The data points are categorized based on the number of markers (horizontal axis) and the inference method (vertical axis) used. Triangles indicate cline widths derived from mitochondrial markers. The red line represents the linear regression line, and the surrounding grey area indicates the 95% confidence interval. The Pearson correlation coefficient (*r*) and its *p*‐value are provided for each plot, with significance denoted as * for *p* < 0.05 and ns for non‐significant (*p* > 0.05). The axes are presented in a log scale.
**Figure S5:** (a) Mean cline widths in kilometres (km) and (b) dispersal‐corrected selection coefficient values plotted against the estimated divergence time of the involved taxa in million years (Ma) for 32 published amphibian hybrid zones. The data points are categorized based on the number of markers (horizontal axis) and the inference method (vertical axis) used. Triangles indicate cline widths derived from mitochondrial markers. The red line represents the linear regression line, and the surrounding grey area indicates the 95% confidence interval. The Pearson correlation coefficient (*r*) and its *p*‐value are provided for each plot, with significance denoted as * for *p* < 0.05 and ns for non‐significant (*p* > 0.05). The axes are presented in a log scale.
**Figure S6:** (a) Mean cline widths in kilometres (km) and (b) dispersal‐corrected selection coefficient values plotted against the estimated divergence time of the involved taxa in million years (Ma) for 12 published mammalian hybrid zones. The data points are categorized based on the number of markers (horizontal axis) and the inference method (vertical axis) used. Triangles indicate cline widths derived from mitochondrial markers. The red line represents the linear regression line, and the surrounding grey area indicates the 95% confidence interval. The Pearson correlation coefficient (*r*) and its *p*‐value are provided for each plot, with significance denoted as * for *p* < 0.05 and ns for non‐significant (*p* > 0.05). The axes are presented in a log scale.
**Figure S7:** (a) Mean cline widths in kilometres (km) and (b) dispersal‐corrected selection coefficient values plotted against the estimated divergence time of the involved taxa in million years (Ma) for 18 published squamate hybrid zones. The data points are categorized based on the number of markers (horizontal axis) and the inference method (vertical axis) used. Triangles indicate cline widths derived from mitochondrial markers. The red line represents the linear regression line, and the surrounding grey area indicates the 95% confidence interval. The Pearson correlation coefficient (*r*) and its *p*‐value are provided for each plot, with significance denoted as * for *p* < 0.05 and ns for non‐significant (*p* > 0.05). The axes are presented in a log scale.
**Figure S8:** (a) Mean cline widths in kilometres (km) and (b) dispersal‐corrected selection coefficient values plotted against the estimated divergence time of the involved taxa in million years (Ma) for 28 published avian hybrid zones. The data points are categorized based on the number of markers (horizontal axis) and the inference method (vertical axis) used. Triangles indicate cline widths derived from mitochondrial markers. The red line represents the linear regression line, and the surrounding grey area indicates the 95% confidence interval. The Pearson correlation coefficient (*r*) and its *p*‐value are provided for each plot, with significance denoted as * for *p* < 0.05 and ns for non‐significant (*p* > 0.05). The axes are presented in a log scale.
**Figure S9:** (a) Mean cline widths in kilometres (km) and (b) dispersal‐corrected selection coefficient values plotted against the estimated divergence time of the involved taxa in million years (Ma) for 13 published hexapodia hybrid zones. The data points are categorized based on the number of markers (horizontal axis) and the inference method (vertical axis) used. Triangles indicate cline widths derived from mitochondrial markers. The red line represents the linear regression line, and the surrounding grey area indicates the 95% confidence interval. The Pearson correlation coefficient (*r*) and its *p*‐value are provided for each plot, with significance denoted as * for *p* < 0.05 and ns for non‐significant (*p* > 0.05). The axes are presented in a log scale.
**Figure S10:** Divergence time estimates in million years (Ma) obtained from literature plotted against the divergence times (Ma) derived from timetree.org for hybridizing species pairs in our dataset for which both estimates are available.
**Table S1:** Primary literature sources for parameter estimates used in this study. For each hybrid zone, the table lists the organismal group, the two interacting lineages, and the original references reporting estimates of cline width, divergence time and dispersal rate. Where available, multiple published estimates were used for a given parameter, and all corresponding source studies are listed within the same column. ‘NA’ indicates that no published estimate was available for the respective parameter.
**Table S2:** Ten best‐fit generalized linear models (GLMs) with a Log‐Gamma distribution, selected based on the corrected Akaike information criterion (AICc), for 71 hybridizing taxa (seen in Figure 1). The models aim to predict the mean cline width (w) in kilometres (km) using the variables: divergence time (T) in million years, taxonomic group (G), and dispersal rate category (D). The weights of the AICc, representing the model's predictive power compared to other models. The first model with lowest AICc was chosen.
**Table S3:** ANOVA of predictors for the best fit‐model. Significance levels: ***p < 0.001, **p < 0.01, *p < 0.05. Df, degree of freedom; Pr(> Chi), *p*‐value associated with the Chi^2^‐test. For categories see this table.
**Table S4:** Predictors and regression values for the best‐fit model. Predictor interactions without occurrences have been omitted for clarity. *N*, number of occurrences. ^1^*p < 0.05; **p < 0.01; ***p < 0.001; ^2^CI, confidence interval; ^3^False discovery rate correction for multiple testing.
**Table S5:** Descriptive statistics of divergence time in million years (Ma) for species pairs obtained from the analysis of seven timetrees from timetree.org.

## Data Availability

All compiled and generated data, code, and results described in this paper are available in the DataDryad repository https://doi.org/10.5061/dryad.wwpzgmsv2. This includes the compiled hybrid zone data, the R scripts for analysis, the source code of the TInflA tool for calculation of divergence time of taxa over time, the timetrees and species lists, and all results produced by TInflA.
