# Supplementary material for: Inferring Time‐to‐Speciation From Hybrid Zone Analysis Informs Assessments of Taxonomic Inflation
Source: Mol Ecol. 2026 May 6;35:e70361. doi: 10.1111/mec.70361 (PMC13147145; doi:10.1111/mec.70361)
Supplement: Supplementary file 1 — Figure S1: The workflow for processing cline widths from hybrid zone literature and incorporating them into the database used in this study. Figure S2: Information criteria (IC) values for all tested models. A red horizontal line denotes the 2 IC units' threshold. Figure S3: Relative importance of the generalized linear model terms across all models. D, dispersal rate category; G, taxonomic group; T, divergence time. Figure S4: (a) Mean cline widths in kilometres (km) and (b) dispersal‐corrected selection coefficient values plotted against the estimated divergence time of the involved taxa in million years (Ma) for all 109 published hybrid zones. The data points are categorized based on the number of markers (horizontal axis) and the inference method (vertical axis) used. Triangles indicate cline widths derived from mitochondrial markers. The red line represents the linear regression line, and the surrounding grey area indicates the 95% confidence interval. The Pearson correlation coefficient (r) and its p‐value are provided for each plot, with significance denoted as * for p < 0.05 and ns for non‐significant (p > 0.05). The axes are presented in a log scale. Figure S5: (a) Mean cline widths in kilometres (km) and (b) dispersal‐corrected selection coefficient values plotted against the estimated divergence time of the involved taxa in million years (Ma) for 32 published amphibian hybrid zones. The data points are categorized based on the number of markers (horizontal axis) and the inference method (vertical axis) used. Triangles indicate cline widths derived from mitochondrial markers. The red line represents the linear regression line, and the surrounding grey area indicates the 95% confidence interval. The Pearson correlation coefficient (r) and its p‐value are provided for each plot, with significance denoted as * for p < 0.05 and ns for non‐significant (p > 0.05). The axes are presented in a log scale. Figure S6: (a) Mean cline widths in kilometres (km) and (b) [file MEC-35-e70361-s001.docx]

Inferring Time-to-Speciation from Hybrid Zone Analysis Informs Assessments of Taxonomic Inflation

Sven Gippner, Katharina Ruthsatz, Christophe Dufresnes, Miguel Vences

Supporting Information

Figures S1-S10

Tables S1-S5

Supplementary references


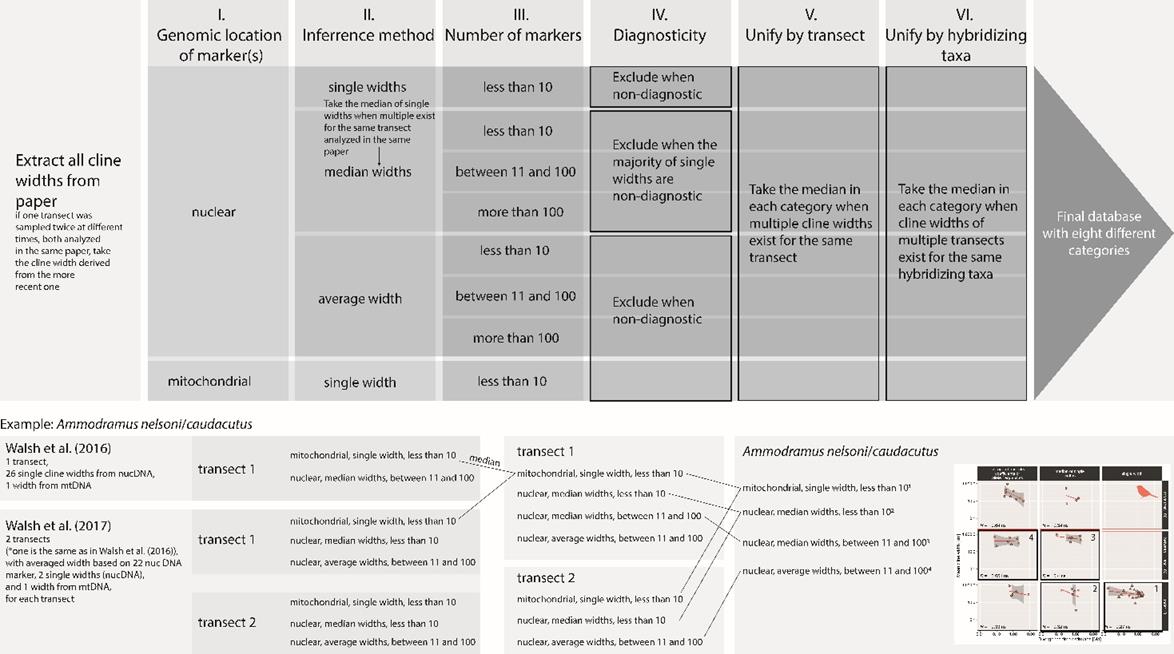


**Figure S1.** The workflow for processing cline widths from hybrid zone literature and incorporating them into the database used in this study.


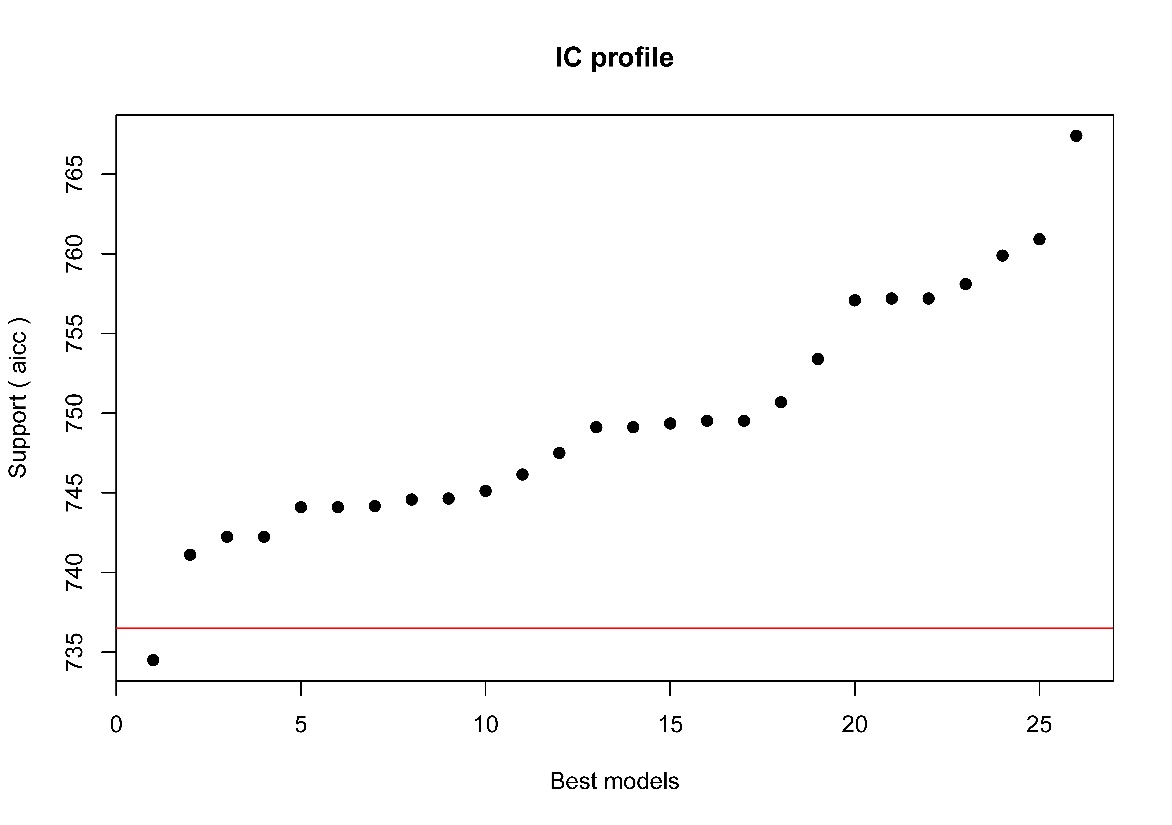


**Figure S2.** Information criteria (IC) values for all tested models. A red horizontal line denotes the 2 IC units’ threshold.


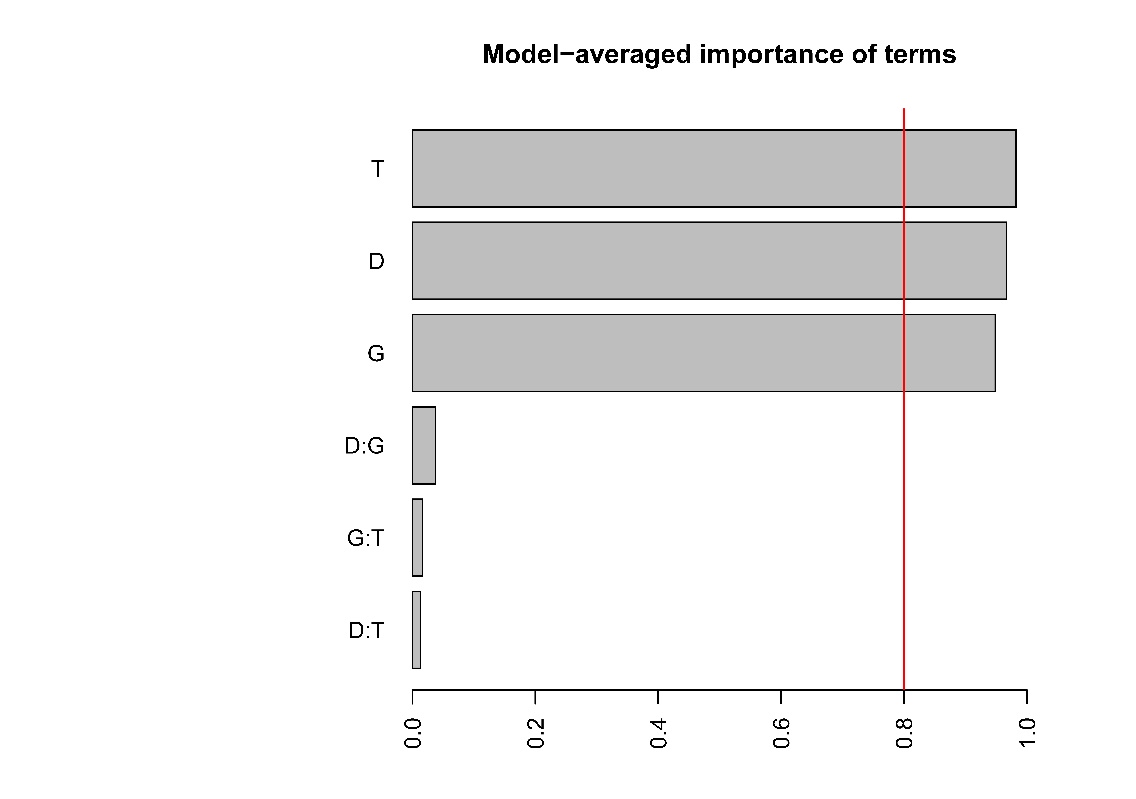


**Figure S3.** Relative importance of the generalized linear model terms across all models. T, divergence time; G, taxonomic group; D, dispersal rate category


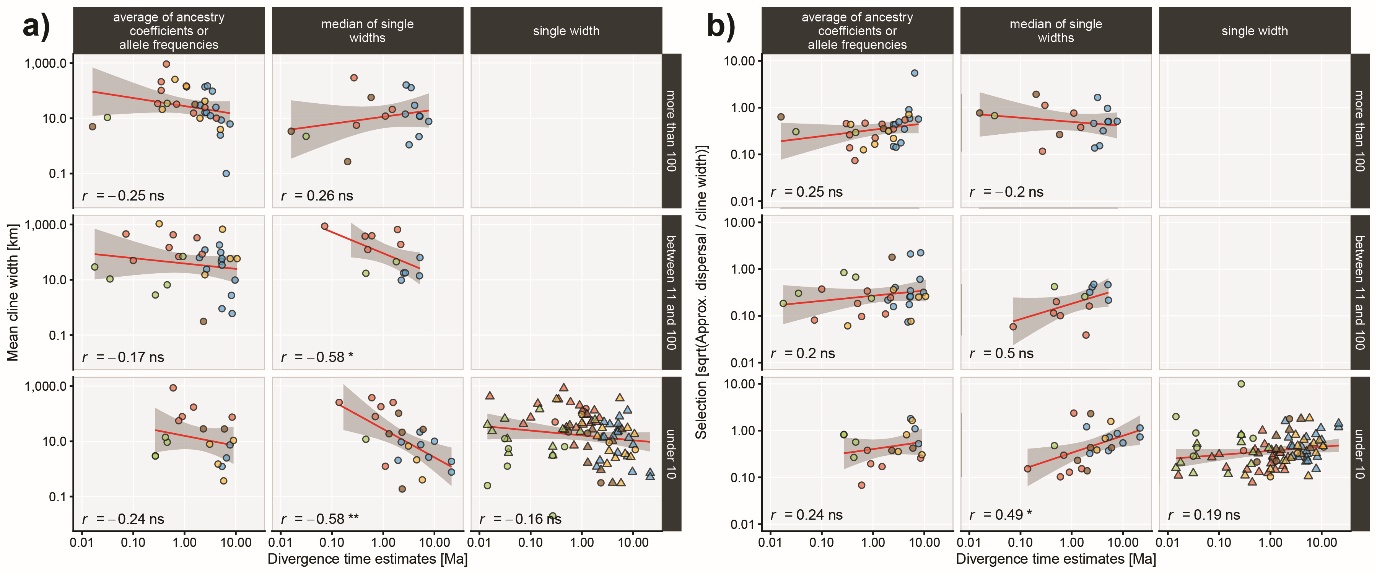


**Figure S4.** (a) Mean cline widths in kilometers (km) and (b) dispersal-corrected selection coefficient values plotted against the estimated divergence time of the involved taxa in million years (Ma) for all 109 published hybrid zones. The data points are categorized based on the number of markers (horizontal axis) and the inference method (vertical axis) used. Triangles indicate cline widths derived from mitochondrial markers. The red line represents the linear regression line, and the surrounding gray area indicates the 95% confidence interval. The Pearson correlation coefficient (r) and its p-value are provided for each plot, with significance denoted as * for p < 0.05 and ns for non-significant (p > 0.05). The axes are presented in a log scale.


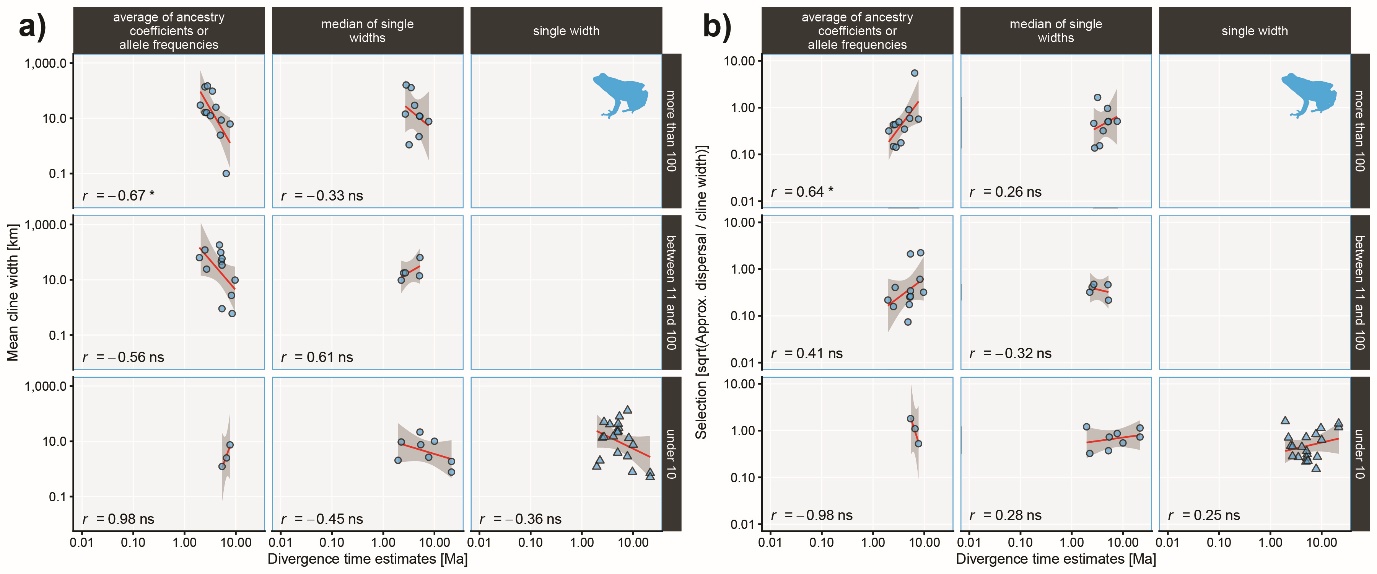


**Figure S5.** (a) Mean cline widths in kilometers (km) and (b) dispersal-corrected selection coefficient values plotted against the estimated divergence time of the involved taxa in million years (Ma) for 32 published amphibian hybrid zones. The data points are categorized based on the number of markers (horizontal axis) and the inference method (vertical axis) used. Triangles indicate cline widths derived from mitochondrial markers. The red line represents the linear regression line, and the surrounding gray area indicates the 95% confidence interval. The Pearson correlation coefficient (r) and its p-value are provided for each plot, with significance denoted as * for p < 0.05 and ns for non-significant (p > 0.05). The axes are presented in a log scale.


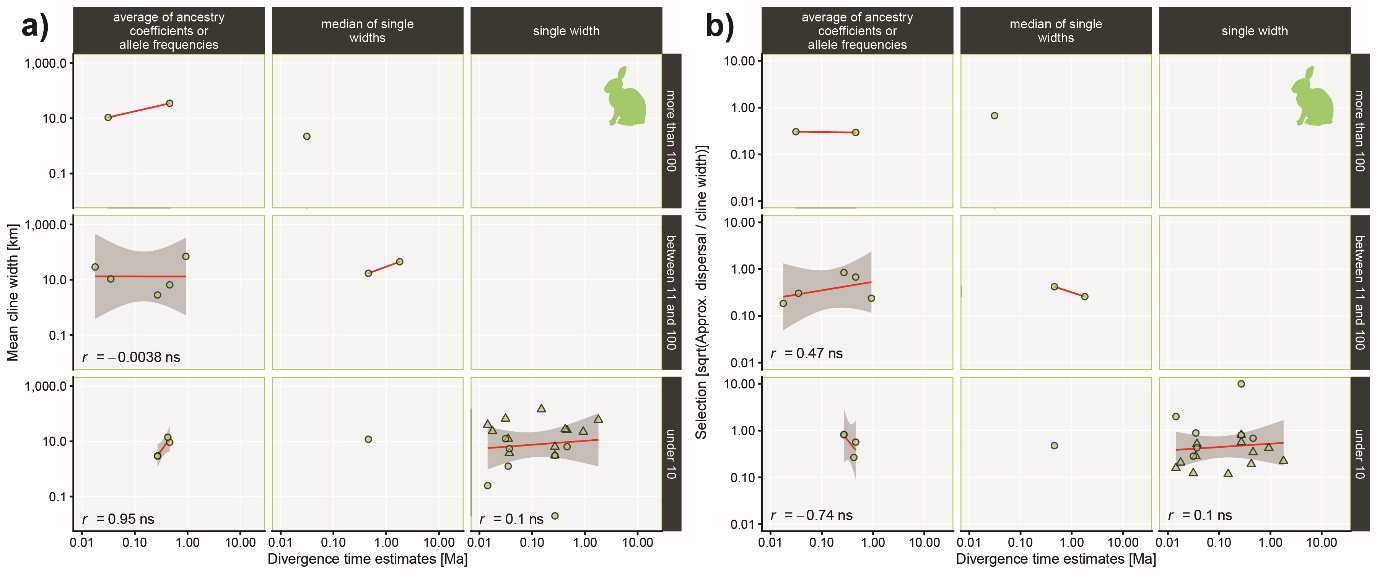


**Figure S6.** (a) Mean cline widths in kilometers (km) and (b) dispersal-corrected selection coefficient values plotted against the estimated divergence time of the involved taxa in million years (Ma) for 12 published mammalian hybrid zones. The data points are categorized based on the number of markers (horizontal axis) and the inference method (vertical axis) used. Triangles indicate cline widths derived from mitochondrial markers. The red line represents the linear regression line, and the surrounding gray area indicates the 95% confidence interval. The Pearson correlation coefficient (r) and its p-value are provided for each plot, with significance denoted as * for p < 0.05 and ns for non-significant (p > 0.05). The axes are presented in a log scale.


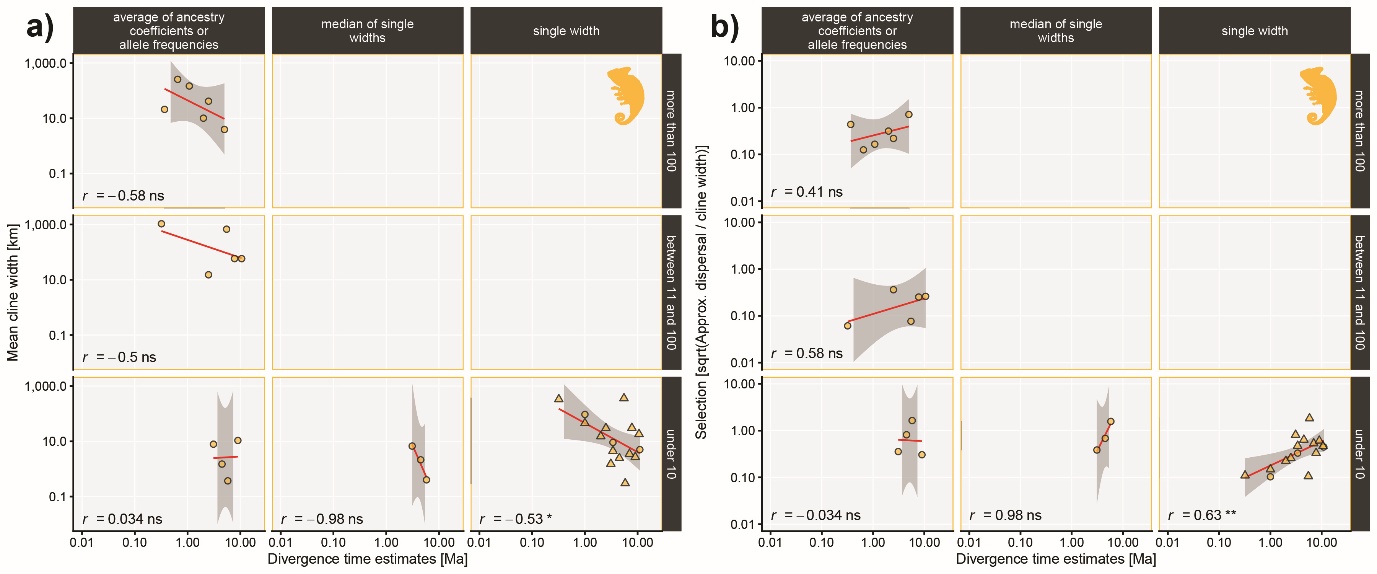


**Figure S7.** (a) Mean cline widths in kilometers (km) and (b) dispersal-corrected selection coefficient values plotted against the estimated divergence time of the involved taxa in million years (Ma) for 18 published squamate hybrid zones. The data points are categorized based on the number of markers (horizontal axis) and the inference method (vertical axis) used. Triangles indicate cline widths derived from mitochondrial markers. The red line represents the linear regression line, and the surrounding gray area indicates the 95% confidence interval. The Pearson correlation coefficient (r) and its p-value are provided for each plot, with significance denoted as * for p < 0.05 and ns for non-significant (p > 0.05). The axes are presented in a log scale.


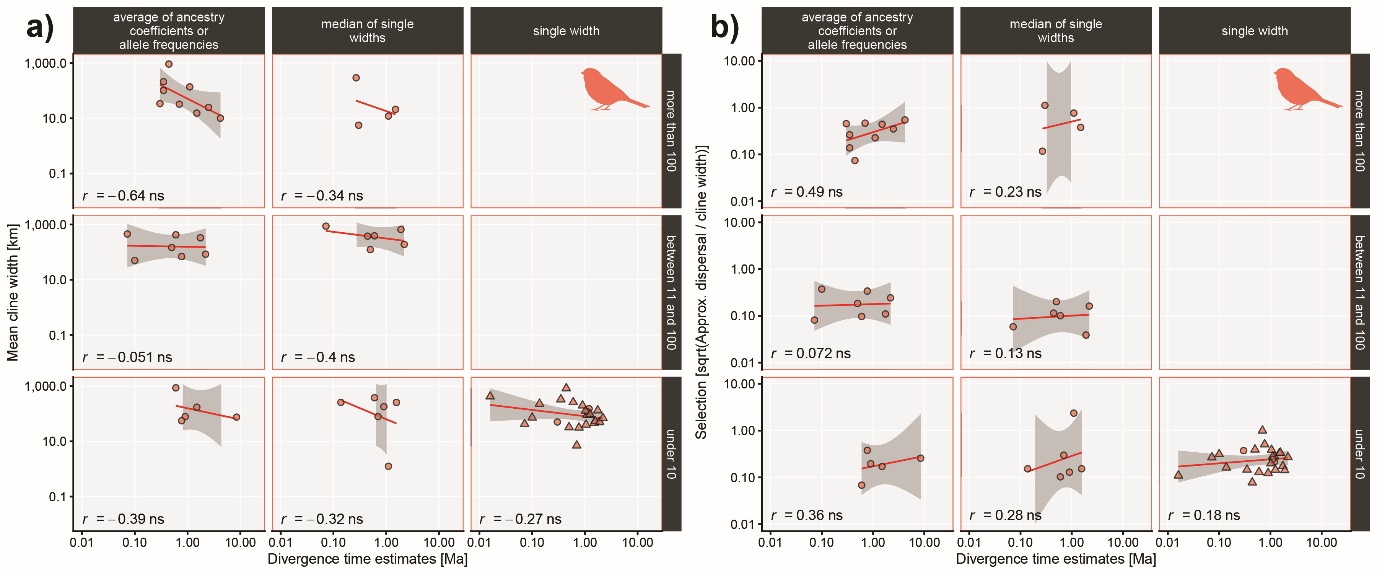


**Figure S8.** (a) Mean cline widths in kilometers (km) and (b) dispersal-corrected selection coefficient values plotted against the estimated divergence time of the involved taxa in million years (Ma) for 28 published avian hybrid zones. The data points are categorized based on the number of markers (horizontal axis) and the inference method (vertical axis) used. Triangles indicate cline widths derived from mitochondrial markers. The red line represents the linear regression line, and the surrounding gray area indicates the 95% confidence interval. The Pearson correlation coefficient (r) and its p-value are provided for each plot, with significance denoted as * for p < 0.05 and ns for non-significant (p > 0.05). The axes are presented in a log scale.


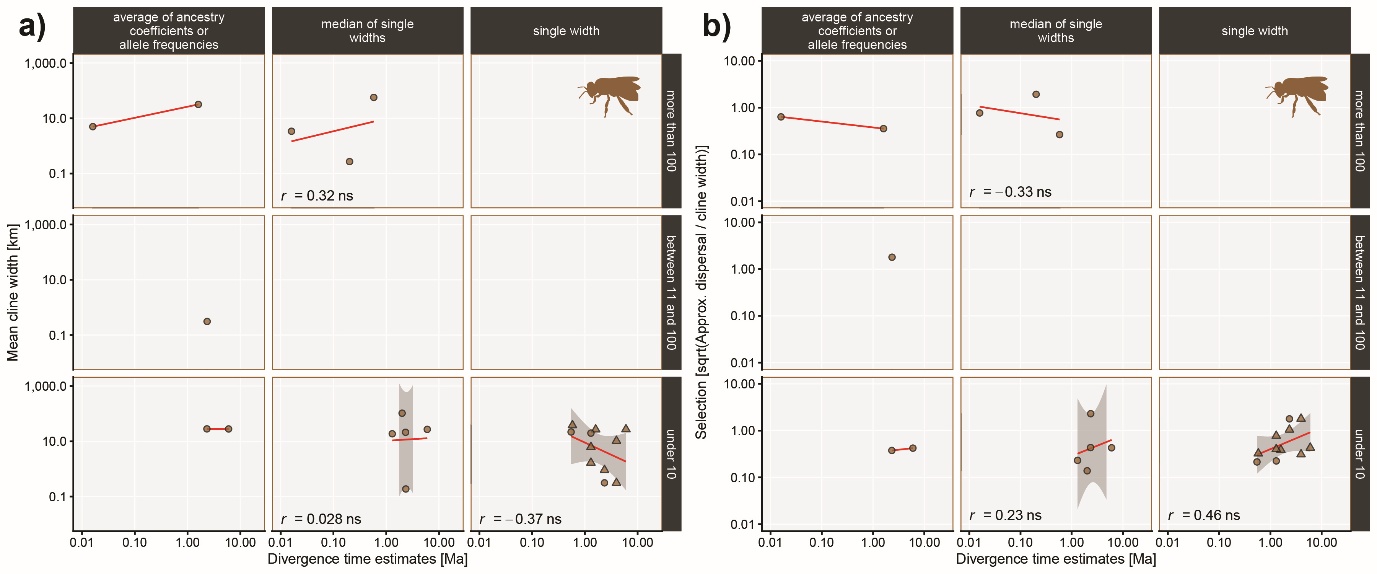


**Figure S9.** (a) Mean cline widths in kilometers (km) and (b) dispersal-corrected selection coefficient values plotted against the estimated divergence time of the involved taxa in million years (Ma) for 13 published hexapodia hybrid zones. The data points are categorized based on the number of markers (horizontal axis) and the inference method (vertical axis) used. Triangles indicate cline widths derived from mitochondrial markers. The red line represents the linear regression line, and the surrounding gray area indicates the 95% confidence interval. The Pearson correlation coefficient (r) and its p-value are provided for each plot, with significance denoted as * for p < 0.05 and ns for non-significant (p > 0.05). The axes are presented in a log scale.


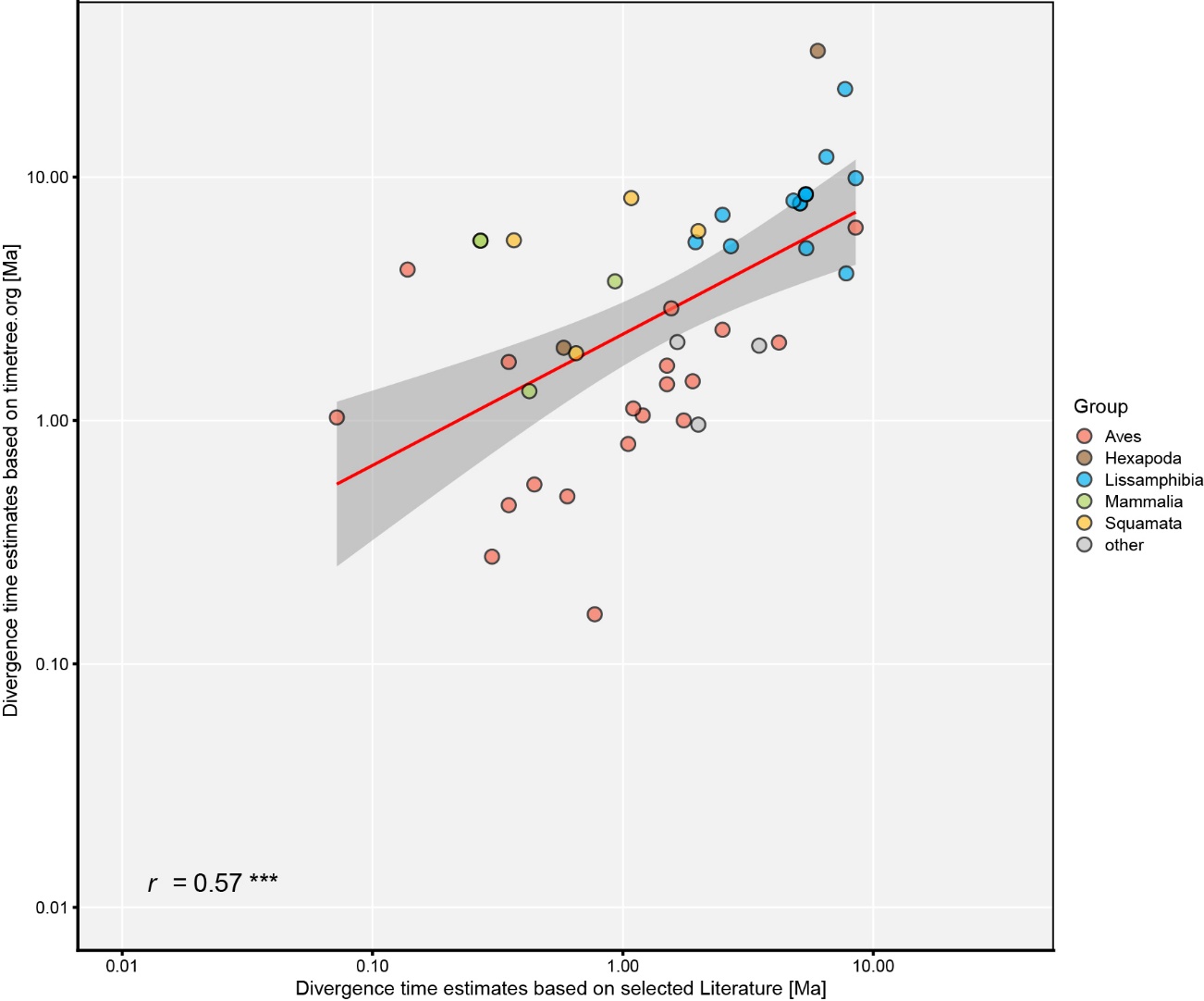


**Figure S10.** Divergence time estimates in million years (Ma) obtained from literature plotted against the divergence times (Ma) derived from timetree.org for hybridizing species pairs in our dataset for which both estimates are available.

**Table S1.** Primary literature sources for parameter estimates used in this study. For each hybrid zone, the table lists the organismal group, the two interacting lineages, and the original references reporting estimates of cline width, divergence time, and dispersal rate. Where available, multiple published estimates were used for a given parameter, and all corresponding source studies are listed within the same column. “NA” indicates that no published estimate was available for the respective parameter.

| **Organismal group** | **Lineage 1** | **Lineage 2** | **Reference(s) cline width** | **Reference(s) divergence time** | **Reference(s) dispersal rate** |
| --- | --- | --- | --- | --- | --- |
| Actinopterygii | *Cottus rhenanus* | *Cottus perifretum* "invasive" | Nolte et al. (2006) | Cheng et al. (2013) | Nolte et al. (2005) |
| Actinopterygii | *Fundulus heteroclitus heteroclitus* | *Fundulus heteroclitus macrolepidotus* | McKenzie et al. (2016), McKenzie et al. (2015) | Gonzalez-Villasenor and Powers (1990) | McKenzie et al. (2015) |
| Actinopterygii | *Fundulus olivaceus* | *Fundulus notatus* | Schaefer et al. (2016) | NA | McKenzie et al. (2015) |
| Actinopterygii | *Gasterosteus aculeatus (stream)* | *Gasterosteus aculeatus (anadromous)* | Vines et al. (2016) | NA | Vines et al. (2016) |
| Actinopterygii | *Nematocharax venustus* (northern) | *Nematocharax venustus* (southern) | Barreto et al. (2020) | Barreto et al. (2022) | Barreto et al. (2016) |
| Actinopterygii | *Symphodus melops* (western) | *Symphodus melops* (south western) | Faust et al. (2021) | Mattingsdal et al. (2020) | Hirase et al. (2020) |
| Anura | *Alytes pertinax* | *Alytes almogavarii* | Dufresnes and Martinez-Solano (2020), Dufresnes et al. (2021) | Goncalves et al. (2015) | Dufresnes et al. (2020) |
| Anura | *Bombina bombina* | *Bombina variegata* | Szymura and Barton (1991); Yanchukov et al. (2006); Hofman and Szymura (2007); Dufresnes et al. (2021a), Dufresnes et al. (2021b) | Pabijan et al. (2013) | Szymura and Barton (1986) |
| Anura | *Bufo bufo* | *Bufo spinosus* | Arntzen et al. (2016); Arntzen et al. (2017); van Riemsdijk et al. (2019); Dufresnes et al. (2020) | Garcia-Porta (2012) | Arntzen et al. (2016) |
| Anura | *Bufotes siculus* | *Bufotes balearicus* | Dufresnes et al. (2021), (Gerchen et al. (2018)) | Dufresnes et al. (2021) | Arntzen et al. (2016) |
| Anura | *Bufotes viridis* | *Bufotes balearicus* | Dufresnes et al. (2014), (Gerchen et al. (2018)), Dufresnes et al. (2021) | Dufresnes et al. (2014) | Arntzen et al. (2016) |
| Anura | *Discoglossus g. galganoi* | *Discoglossus g. jeanneae* | Dufresnes et al. (2020), Dufresnes et al. (2021) | Dufresnes et al. (2021) | Dufresnes et al. (2020) |
| Anura | *Discoglossus pictus* | *Discoglossus scovazzi* | Dufresnes et al. (2020), Dufresnes et al. (2021) | Dufresnes et al. (2021) | Dufresnes et al. (2020) |
| Anura | *Hyla arborea* | *Hyla orientalis* | Dufresnes et al. (2015), Dufresnes et al. (2021) | Dufresnes et al. (2021) | Dufresnes et al. 2015 |
| Anura | *Hyla arborea* | *Hyla molleri* | Dufresnes et al. (2020), Dufresnes et al. (2021) | Dufresnes et al. (2020) | Dufresnes et al. (2020) |
| Anura | *Hyla perrini* | *Hyla intermedia* | Dufresnes et al. (2018), Dufresnes et al. (2021) | Dufresnes et al. (2018) | Dufresnes et al. (2018) |
| Anura | *Hyla perrini* | *Hyla arborea* | Dufresnes et al. (2021), (Verardi et al. (2009)) | Dufresnes et al. (2021) | Dufresnes et al. (2020) |
| Anura | *Litoria ewingii* | *Litoria paraewingi* | Smith et al. (2013a), Smith et al. (2013b) | NA | Smith et al. (2013) |
| Anura | *Odorrana margaretae* (north) | *Odorrana margaretae* (west) | Wen and Fu (2021) | Wen and Fu (2021) | Berven and Grudzien (1990) |
| Anura | *Pelobates fuscus* | *Pelobates vespertinus* | Dufresnes et al. (2019), Dufresnes et al. (2021) | Dufresnes et al. (2019) | Dufresnes et al. (2019) |
| Anura | *Pelodytes ibericus* | *Pelodytes atlanticus* | Dufresnes et al. (2020), Dufresnes et al. (2021) | Dufresnes et al. (2020) | Dufresnes et al. (2020) |
| Anura | *Pelodytes punctatus* | *Pelodytes hespericus* | Dufresnes et al. (2020), Dufresnes et al. (2021) | Dufresnes et al. (2020) | Dufresnes et al. (2020) |
| Anura | *Pelophylax bergeri* north | *Pelophylax bergeri* south | Santucci et al. (1996), Dufresnes et al. (2021) | Santucci (1996) | Berven and Grudzien (1990) |
| Anura | *Pseudacris crucifer* Interior | *Pseudaris crucifer* Eastern | Stewart et al. (2016) | Steward et al. (2016) | Lemmon and Lemmon (2008) |
| Anura | *Pseudacris nigrita* | *Pseudacris fouquettei* | Engebretsen et al. (2016) | Lemmon et al. (2007) | Lemmon and Lemmon (2008) |
| Anura | *Rana berlandieri* | *Rana utricularia (sphenocephala)* | Kocher and Sage (1986) | NA | Berven and Grudzien (1990) |
| Anura | *Rana chensinensis* | *Rana kukunoris* | Qi et al. (2014) | Zhou et al. (2012) | Berven and Grudzien (1990) |
| Anura | *Rana temporaria* | *Rana parvipalmata* | Dufresnes et al. (2020), Dufresnes et al. (2021) | Dufresnes et al. (2020) | Berven and Grudzien (1990) |
| Aves | *Alectoris rufa* | *Alectoris graeca* | Randi and Bernard-Laurent (1999) | Seabrook-Davison et al. (2009) | Bernard-Laurent (1991) & Bernard-Laurent (1991) [could only found one] |
| Aves | *Ammodramus nelsoni* | *Ammodramus caudacutus* | Walsh et al. (2016); Walsh et al. (2017) | Rising and Avise (1993) | Walsh et al. (2012), Gjerdrum et al. (2008) |
| Aves | *Aphelocoma californica californica* | *Aphelocoma californica woodhousei* | Gowen et al. (2014) | McCormack et al. (2011) | Carmen (1988) |
| Aves | *Catharus ustulatus ustalutus* | *Catharus ustulatus swainsoni* | Ruegg (2008) | Ruegg and Smith (2002) | Ruegg (2008) |
| Aves | *Cinnyris moreaui* | *Cinnyris fuelleborni* | McEntee et al. (2016) | NA | Haran et al. (2018) |
| Aves | *Corvus caurinus* | *Corvus brachyrhynchos* | Slager et al. (2020) | Slager et al. (2020) | Paradis et al. (1998) |
| Aves | *Dinopium benghalense* | *Dinopium psarodes* | Fernando et al. (2016) | NA | Paradis et al. 1998 |
| Aves | *Empidonax difficilis* | *Empidonax occidentalis* | Linck et al. (2019) | Rush et al. (2009) | Mathewson et al. (2013) |
| Aves | *Empidonax occidentalis* MXO | *Empidonax occidentalis* MXS | Linck et al. (2019) | NA | Mathewson et al. (2013) |
| Aves | *Empidonax occidentalis* USW | *Empidonax occidentalis* MXO | Linck et al. (2019) | NA | Mathewson et al. (2013) |
| Aves | *Hirundo rustica rustica* | *Hirundo rustica tytleri* | Scordato et al. (2017); Scordato et al. (2020); Schield et al. (2021) | Zink et al. (2014) | Scordato et al. (2020) |
| Aves | *Hirundo rustica rustica* | *Hirundo rustica gutturalis* | Scordato et al. (2020); Schield et al. (2021) | Zink et al. (2014) | Scordato et al. (2020) |
| Aves | *Hirundo rustica tytleri* | *Hirundo rustica gutturalis* | Scordato et al. (2017); Scordato et al. (2020); Schield et al. (2021) | Scordato et al. (2017) | Scordato et al. (2020) |
| Aves | *Hypocnemis ochrogyna* | *Hypocnemis striata* | Cronemberger et al. (2020) | Cronemberger et al. (2020) | Cronemberger et al. (2020) |
| Aves | *Icterus galbula* | *Icterus bullockii* | Carling et al. (2011); Walsh et al. (2020) | Jacobsen and Omland (2012) | Moore and Dolbeer (1989) |
| Aves | *Jacana spinosa* | *Jacana jacana* | Miller et al. (2014); Lipshutz et al. (2018) | Miller et al. (2014) | Jackson et al. (2019) |
| Aves | *Larus glaucescens* | *Larus occidentalis* | Gay et al. (2008) | NA | Gay et al. (2008), Paradis et al. (1998) |
| Aves | *Malurus melanocephalus cruentatus* | *Malurus melanocephalus melanocephalus* | Baldassare et al. (2014) | Lee and Edwards (2008) | Baldassare et al. (2014) |
| Aves | *Manacus vitellinus* | *Manacus candei* | Yuri et al. (2009), (Brumfield et al. 2001) | NA | Snow and Lill (1974) |
| Aves | *Motacilla alba alba* | *Motacilla alba personata* | Semenov et al. (2017) | Li et al. (2016) | Paradis et al. (1998) |
| Aves | *Myioborus ornatus* | *Myioborus melanocephalus* | Cespedes-Arias et al. (2021) | Cespedes-Arias et al. (2021) | Cespedes-Arias et al. (2021) |
| Aves | *Oporornis tolmiei* | *Oporornis philadelphia* | Irwin et al. (2009) | Weir and Schluter (2004) | Erickson et al. (2008) |
| Aves | *Passer italiae* | *Passer domesticus* | Bailey et al. (2015) | Päckert et al. (2021) | Anderson 2006, Bailey et al. (2015) |
| Aves | *Passerina amoena* | *Passerina cyanea* | Carling and Brumfield (2008); Carling and Zuckerberg (2011) | Carling and Brumfield (2008) | Carling and Brumfield (2008) |
| Aves | *Periparus ater melanolophus* | *Periparus ater aemodius* | Wolfgramm et al. (2021) | Wolfgramm et al. (2021) | Paradis et al. (1998) |
| Aves | *Pheucticus melanocephalus* | *Pheucticus ludovicianus* | Mettler and Spellman (2009) | Pulgarin-R et al. (2013) | Carling and Brumfield (2008) |
| Aves | *Phylloscopus trochilus* S | *Phylloscopus trochilus* N | Bensch et al. (2009) | Bensch et al. (2009) | Paradis et al. (1998) |
| Aves | *Pipilo ocai* | *Pipilo maculatus* | Kingston et al. (2012) | NA | Lehnen and Rodewald (2009), Paradis et al. 1998 |
| Aves | *Poecile atricapillus* | *Poecile carolinensis* | Taylor et al. (2014), Wagner et al. (2020) | NA | Paradis et al. (1998) |
| Aves | *Poephila acuticauda acuticauda* | *Poephila acuticauda hecki* | Lopez et al. (2021) | Lopez et al. (2021) | Zann and Runciman (1994) |
| Aves | *Rhegmatorhina hoffmansi* | *Rhegmatorhina berlepschi* | Del-Rio et al. (2021) | Del-Rio et al. (2021) | Del-Rio et al. (2021) |
| Aves | *Setophaga auduboni* | *Setophaga coronata* | Brelsford and Irwin (2009) | Mila et al. (2007) | Brelsford and Irwin (2009) |
| Aves | *Setophaga auduboni* N | *Setophaga auduboni* S | Toews et al. (2014); Mila et al. (2011) | Mila et al. (2011) | Brelsford and Irwin (2009) |
| Aves | *Setophaga townsendi* | *Setophaga virens* | Toews et al. (2011) | Weir and Schluter (2004) | Rohwer and Wood (1998) |
| Aves | *Sphyrapicus ruber* | *Sphyrapicus nuchalis* | Grossen et al. (2016); Seneviratne et al. (2016) | Weir and Schluter (2004) | Moore and Buchanan (1985) |
| Aves | *Sphyrapicus ruber* | *Sphyrapicus varius* | Grossen et al. (2016); Seneviratne et al. (2016); Seneviratne et al. (2012) | Weir and Schluter (2004) | Moore and Buchanan (1985) |
| Aves | *Sphyrapicus varius* | *Sphyrapicus nuchalis* | Natola et al. (2021) | Weir and Schluter (2004) | Moore and Buchanan (1985) |
| Aves | *Strix occidentalis caurina* | *Strix occidentalis occidentalis* | Barrowclough et al. (2005) | Haig et al. (2004) | Paradis et al. (1998) |
| Aves | *Sula nebouxii* | *Sula variegata* | Taylor et al. (2012) | Patterson et al. (2010) | Osorio-Beristain and Drummond (1993) |
| Aves | *Synallaxis ruficapilla* | *Synallaxis cinerea* | Batalha-Filho et al. (2019) | Batlha-Filho et al. (2019) | Batalha-Filho et al. (2019) |
| Aves | *Thamnophilus caerulescens aspersiventer* | *Thamnophilus caerulescens dinellii* | Brumfield (2005) | Brumfield (2005) | Tarwater (2012) |
| Aves | *Vireo gilvus gilvus* | *Vireo gilvus swainsoni* | Lovell et al. (2021) | Lovell et al. (2021) | Lovell et al. (2021) |
| Aves | *Willisornis poecilinotus* | *Willisornis vidua* | Pulido-Santacruz et al. (2018) | Weir et al. (2015), citation from supplements of Pulido-Santacruz et al. (2018) | Pulido-Santacruz (2018) |
| Aves | *Xiphorhynchus elegans* | *Xiphorhynchus spixii* | Pulido-Santacruz et al. (2018) | Weir et al. (2015), citation from supplements of Pulido-Santacruz et al. (2018) | Pulido-Santacruz (2018) |
| Aves | *Zonotrichia capensis (not elevated)* | *Zonotrichia capensis (elevated)* | Cheviron and Brumfield (2009) | NA | Cheviron and Brumfield (2009) |
| Aves | *Zosterops pallidus* | *Zosterops virens* | Oatley et al. (2017) | Oatley et al. (2012) | Moyle et al. (2009) |
| Caudata | *Ambystoma maculatum West* | *Ambystoma maculatum East* | Johnson et al. (2015) | Zamudio and Savage (2003) | Arntzen and Wallis (1991) |
| Caudata | *Chioglossa lusitanica longipes* | *Chioglossa lusitanica lusitanica* | Sequeira et al. (2005); Sequeira et al. (2022) | Alexandrino et al. (2000) | Arntzen (1980) |
| Caudata | *Cynops pyrrhogaster Central* | *Cynops pyrrhogaster Western* | Tominaga et al. (2018) | Tominaga et al. (2013) | Tominaga et al. (2021) |
| Caudata | *Cynops pyrrhogaster Central* | *Cynops pyrrhogaster Northern* | Tominaga et al. (2021) | Tominaga et al. (2013) | Tominaga et al. (2021) |
| Caudata | *Ensatina eschscholtzii eschscholtzii* | *Ensatina eschscholtzii klauberi* | Devitt et al (2011) | Kutcha et al. (2009) | Alexandrino et al. (2005) |
| Caudata | *Ensatina eschscholtzii xanthoptica* | *Ensatina eschscholtzii platensis* | Alexandrino et al. (2005) | Kutcha et al. (2009) | Alexandrino et al. (2005) |
| Caudata | *Lissotriton boscai* | *Lissotriton maltzani* | Sequeira et al. (2020) | NA | Sequeira et al. (2020) |
| Caudata | *Lissotriton montandoni* | *Lissotriton vulgaris* | Zielinski et al. (2019) | Zielinski et al. 2016 | Smith and Green (2005) |
| Caudata | *Taricha torosa torosa* | *Taricha torosa sierrae* | Kuchta (2007) | Kuchta and Tan (2006) | Kuchta (2007) |
| Caudata | *Triturus anatolicus* | *Triturus ivanbureschi* | Wielstra et al. (2017) | Ehl et al. (2019) | Wielstra et al. (2017) |
| Caudata | *Triturus cristatus* | *Triturus carnifex* | Macat et al. (2019) | supplements of Ehl et al. (2019) | Wielstra et al. (2017) |
| Caudata | *Triturus cristatus* | *Triturus ivanbureschi* | Wielstra et al. (2017) | supplements of Ehl et al. (2019) | Wielstra et al. (2017) |
| Caudata | *Triturus dobrogicus* | *Triturus ivanbureschi* | Wielstra et al. (2017) | supplements of Ehl et al. (2019) | Wielstra et al. (2017) |
| Caudata | *Triturus macedonicus* | *Triturus ivanbureschi* | Wielstra et al. (2017) | supplements of Ehl et al. (2019) | Wielstra et al. (2017) |
| Caudata | *Triturus marmoratus* | *Triturus pygmaeus* | Arntzen (2018) | Arntzen et al. (2007) | Wielstra et al. (2017) |
| Cnidaria | *Eunicea flexuosa* lineage 1 | *Eunicea flexuosa* lineage 2 | Prada and Hellberg (2014) | Prada and Hellberg (2021) | Prada and Hellberg (2014) |
| Crocodylia | *Crocodylus moreletti* | *Crocodylus acutus* | Pacheco-Sierra et al. (2016) | Pacheco-Sierra et al. 2018 | Pacheco-Sierra et al. (2016) |
| Crustacea | *Menippe mercenaria* | *Menippe adina* | Bert and Harrison (1988) | Seyoum et al. (2021) | Ehrhardt et al. (1990) |
| Hexapoda | *Anartia fatima* | *Anartia amathea* | Dasmahapatra et al. (2002) | Wahlberg (2006) | Dasmahapatra et al. (2002) |
| Hexapoda | *Carabus lewisianus* | *Carabus albrechti* | Takami and Suzuki (2005) | NA | Welling (1990) [only abstract available]; Den Boer (1970) |
| Hexapoda | *Chorthippus parallelus parallelus* | *Chorthippus parallelus erythropus* | Vazquez et al. (1994) | Lunt et al. (1998) | Virdee and Hewitt (1994) |
| Hexapoda | *Coenonympha macromma* | *Coenonympha gardetta* | Capblancq et al. (2020) | Capblancq et al. (2020) | Binzenhöfer et. al. (2005) |
| Hexapoda | *Gryllus pennsylvanicus* | *Gryllus firmus* | Larson et al. (2013) | Maroja et al. (2009) | Larson et al. (2013) |
| Hexapoda | *Heliconius erato* E3 | *Heliconius erato* not-E3 | Hill et al. (2013) | Kozak et al. (2015) | Mallet et al. (1990) |
| Hexapoda | *Heliconius erato emma* | *Heliconius erato favorinus* | Mallet et al. (1990) | NA | Mallet et al. (1990) |
| Hexapoda | *Heliconius melpomene amaryllis* | *Heliconius melpomene aglaope* | Mallet et al. (1990) | NA | Mallet et al. (1990) |
| Hexapoda | *Hemideina thoracica* 15 | *Hemideina thoracica* 17 | Morgan-Richards et al. (2000), Morgan-Richards and Wallis (2003) | Morgan-Richards et al. (2003) | Morgan-Richards et al. (2000) |
| Hexapoda | *Hemideina thoracica* 17 | *Hemideina thoracica* 15' | Morgan-Richards and Wallis (2003) | Morgan-Richards et al. (2003) | Morgan-Richards et al. (2000) |
| Hexapoda | *Hemideina thoracica* 17' | *Hemideina thoracica* 19 | Morgan-Richards and Wallis (2003) | Morgan-Richards et al. (2003) | Morgan-Richards et al. (2000) |
| Hexapoda | *Hemideina thoracica* 19 | *Hemideina thoracica* 17 | Morgan-Richards and Wallis (2003) | Morgan-Richards et al. (2003) | Morgan-Richards et al. (2000) |
| Hexapoda | *Hemideina thoracica* 23 | *Hemideina thoracica* 19 | Morgan-Richards and Wallis (2003) | Morgan-Richards et al. (2003) | Morgan-Richards et al. (2000) |
| Hexapoda | *Lycaeides idas* | *Lycaeides melissa* | Gompert et al. (2010) | Talavera et al. (2012) | Knutson et al. (1999) |
| Hexapoda | *Orchelimum nigripes* | *Orchelimum pulchellum* | Shapiro (1998) | NA | Shapiro (1998) |
| Hexapoda | *Papilio glaucus* | *Papilio canadensis* | Ryan et al. (2017) | Kunte et al. (2011) | Lederhouse (1983) |
| Hexapoda | *Pontia daplidice* | *Pontia edusa* | Porter et al. (1997) | Ebdon et al. (2021) | Porter et al. (1997) |
| Hexapoda | *Vandiemenella viatica* P24(XY) | *Vandiemenella viatica* 17 | Kawakami et al. (2009) | Kawakami et al. (2007) | Kawakami et al. (2009) |
| Hexapoda | *Zootermopsis nevadensis nuttingi* | *Zootermopsis nevadensis nevadensis* | Aldrich and Kambhampati (2009) | Aldrich and Kambhampati (2009) | Aldrich and Kambhampati (2009) |
| Mammalia | *Eulemur rufifrons* | *Eulemur cinereiceps* | Delmore et al. (2013) | Marklof and Kappeler (2013) | Delmore et al. (2013) |
| Mammalia | *Geomys bursarius* | *Geomys lutescens* | Heaney and Timm (1985) | NA | Lidicker and Patton (1987) |
| Mammalia | *Microtus arvalis* C | *Microtus arvalis* Italian | Sutter et al. (2013) | Heckel et al. (2005) | Lidicker and Patton (1987) |
| Mammalia | *Microtus arvalis* E | *Microtus arvalis* C | Beysard and Heckel (2014) | Heckel et al. (2005) | Lidicker and Patton (1987) |
| Mammalia | *Microtus arvalis* W | *Microtus arvalis C* | Beysard and Heckel (2014); (Sutter et al. 2013) | Heckel et al. (2005) | Lidicker and Patton (1987) |
| Mammalia | *Microtus arvalis* W | *Microtus arvalis* Italian | Sutter et al. (2013) | Heckel et al. (2005) | Lidicker and Patton (1987) |
| Mammalia | *Microtus californicus* N | *Microtus californicus* S | Conroy and Gupta (2011); Lin et al. (2018) | Lin et al. (2018) | Lidicker and Patton (1987) |
| Mammalia | *Mus musculus musculus* | *Mus musculus domesticus* | Bozikova et al. (2005), Dod et al. (2005), Dufkova et al. (2011), Macholan et al. (2007), Payseur et al. (2004), Payseur and Nachman (2005), Raufaste et al. (2005), Teeter et al. (2008), Teeter et al. (2010), Tucker et al. (1992), Wang et al. (2011) | Suzuki et al. (2013) | Macholan et al. (2007) |
| Mammalia | *Myodes glareolus,* Eastern | *Myodes glareolus,* Carpathian | Tarnowska et al. (2016) | Hornikova et al. (2021) | Kozakiewicz et al. (2007) |
| Mammalia | *Neotoma floridana* | *Neotoma micropus* | Mauldin et al. (2021) | Ordonez-Garca et al. (2014) | Mauldin et al. (2021) |
| Mammalia | *Neotoma lepida* | *Neotoma bryanti* | Jahner et al. (2021) | Jahner et al. (2021) | Mauldin et al. (2021) |
| Mammalia | *Oryctolagus cuniculus algirus* | *Oryctolagus cuniculus cuniculus* | Carneiro et al. (2013); Alda and Doadrio (2014); Rafati et al. (2018) | Carneiro et al. (2009) | Richardson et al. (2002), Carneiro et al. (2013) |
| Mammalia | *Sorex araneus Cordon* | *Sorex antinorii* | Yannic et al. (2008) | Yannic et al. (2008) | Polyakov et al. (2011) |
| Mammalia | *Sorex araneus Vaud* | *Sorex antinorii* | Yannic et al. (2009); Yannic et al. (2008) | Yannic et al. (2008) | Polyakov et al. (2011) |
| Mammalia | *Tamiasciurus douglasii* | *Tamiasciurus hudsonicus* | Chavez et al. (2011) | Chavez et al. (2013) | Sun (1997) |
| Mammalia | *Thomomys bottae connectans* | *Thomomys bottae opulentus* | Smith et al. (1983) | NA | Vaughan (1963) |
| Mollusca | *Arion vulgaris* | *Arion rufus* | Zemanova et al. 2017 | Quinteiro et al. (2005) | Grimm and Paill (2001) |
| Mollusca | *Crassostrea virginica* Atlantic | *Crassostrea virginica* Gulf | Hare and Avise (1996) | Reeb and Avise (1990) | Rose et al. (2006) |
| Mollusca | *Mytilus edulis* | *Mytilus trossulus* | Stuckas et al. (2009) | Riginos and Henzler (2008) | Gilg and Hilbish (2003) |
| Mollusca | *Patella rustica* C | *Patella rustica* D | Sa-Pinto et al. (2010) | NA | Ribeiro (2008) |
| Mollusca | *Placopecten magellanicus* (north) | *Placopecten magellanicus* (south) | Van Wyngaarden et al. (2017) | NA | Van Wyngaarden et al. (2017) |
| Squamata | *Ameiva chrysolaema boekeri* | *Ameiva chrysolaema procax* | Gifford (2008) | Gifford and Larson (2008) | Dessauer et al. (2000) |
| Squamata | *Anolis distichus ignigularis* | *Anolis distichus dominicensis* | Case and Williams (1984) | Geneva et al. (2015) | Calsbeek et al. (2014) |
| Squamata | *Anolis roquet* NW | *Anolis roquet* C | Johansson et al. (2008) | Thorpe et al. (2008) | Calsbeek et al. (2014) |
| Squamata | *Aspidoscelis [tigris] punctilinealis* | *Aspidoscelis marmoratus* | Dessauer et al. (2000) | NA | Dessauer et al. 2000 |
| Squamata | *Carlia rubrigularis* N | *Carlia rubrigularis* S | Phillips et al. (2004); Singhal and Moritz (2013) | Singhal and Moritz (2013) | Phillips et al. (2004) |
| Squamata | *Hoplodactylus maculatus* | *Hoplodactylus maculatus* | Fitness et al. (2011) | NA | Fitness et al. (2011) |
| Squamata | *Lacerta nevadensis* | *Lacerta lepida* | Miraldo et al. (2012) | Miraldo et al. (2012) | Vercken et al. 2012 |
| Squamata | *Lampropholis coggeri* C | *Lampropholis coggeri* S | Singhal and Moritz (2013) | Singhal and Moritz (2013) | Phillips et al. (2004) |
| Squamata | *Lampropholis coggeri* N | *Lampropholis coggeri* C | Singhal and Moritz (2013) | Singhal and Moritz (2013) | Phillips et al. (2004) |
| Squamata | *Liolaemus melanops* | *Liolaemus shehuen* | Grummer et al. (2021) | NA | Camargo et al. (2013) |
| Squamata | *Liolaemus melanops* | *Liolaemus xanthoviridis* | Grummer et al. (2021) | NA | Camargo et al. (2013) |
| Squamata | *Liolaemus xanthoviridis* | *Liolaemus fitzingerii* | Grummer et al. (2021) | NA | Camargo et al. (2013) |
| Squamata | *Natrix astreptophora* | *Natirx helvetica* | Asztalos et al. (2020) | Kindler et al. (2018) | Burbrink et al. (2020) |
| Squamata | *Natrix helvetica* | *Natrix natrix* | Kindler et al. (2017); Schultze et al. (2019) | Fritz et al. (2012) | Burbrink et al. (2020) |
| Squamata | *Natrix helvetica* (northwest) | *Natrix helvetica* (south) | Schultze et al. (2020) | Kindler et al. (2018) | Burbrink et al. (2020) |
| Squamata | *Natrix natrix* | *Natrix tessellata* | Asztalos et al. (2021), Schultze et al. (2020) | Guo et al. (2012) | Burbrink et al. (2020) |
| Squamata | *Natrix natrix vulgaris* (red lineage) | *Natrix natrix natrix* (yellow lineage) | Kindler et al. (2017) | Fritz et al. (2012) | Burbrink et al. (2020) |
| Squamata | *Pantherophis alleghaniensis* | *Pantherophis spiloides* | Burbink et al. (2020) | Burbrink et al. (2020) | Burbrink et al. (2020) |
| Squamata | *Pantherophis obsoletus* | *Pantherophis bairdi* | Burbink et al. (2020) | Burbrink et al. (2020) | Burbrink et al. (2020) |
| Squamata | *Pantherophis spiloides* | *Pantherophis obsoletus* | Burbink et al. (2020) | Burbrink et al. (2020) | Burbrink et al. (2020) |
| Squamata | *Podarcis guadarramae guadarramae* | *Podarcis guadarramae lusitanica* | Caeiro-Dias et al. (2021) | Caeiro-Dias et al. (2021) | Scaletool website: http://scales.ckff.si/scaletool/index.php?menu=6&submenu=0&sid=113 |
| Squamata | *Podarcis muralis* Italian | *Podarcis muralis* Western European | While et al. (2015), Yang et al. (2020) | Yang et al. (2020) | Scaletool website: http://scales.ckff.si/scaletool/index.php?menu=6&submenu=0&sid=113 |
| Squamata | *Saproscincus basiliscus* C | *Saproscincus basiliscus* N | Singhal and Moritz (2013) | Singhal and Moritz (2013) | Phillips et al. (2004) |
| Squamata | *Sceloporus cowlesi* | *Sceloporus tristichus* | Leache et al. (2017), Leache and Cole (2007) | Leache et al. 2016 | Massot et al. (2003) |
| Squamata | *Sceloporus grammicus* F5 | *Sceloporus grammicus* FM2 | Marshall and Sites (2001) | NA | Sites et al. (1995) |
| Testudinata | *Emys orbicularis galloitalica* | *Emys orbicularis hellenica* | Vamberger et al. (2015) | NA | Molnar et al. (2011) |
| Testudinata | *Emys orbicularis orbicularis* | *Emys orbicularis occidentalis* | Pöschel et al. (2018) | NA | Molnar et al. (2011) |
| Testudinata | *Emys trinacris* | *Emys orbicularis* | Vamberger et al. (2015) | NA | Molnar et al. (2011) |

**Table S2.** Ten best-fit generalized linear models (GLMs) with a Log-Gamma distribution, selected based on the corrected Akaike information criterion (AICc), for 71 hybridizing taxa (seen in Figure 1). The models aim to predict the mean cline width (w) in kilometers (km) using the variables: divergence time (T) in million years, taxonomic group (G), and dispersal rate category (D). The weights of the AICc, representing the model's predictive power compared to other models. The first model with lowest AICc was chosen.

| Model | AICc | Weights |
| --- | --- | --- |
| w ~ 1 + D + T + G | 734.494328 | 0.886574 |
| w ~ 1 + D + T | 741.1144539 | 0.032372 |
| w ~ 1 + T + G + D:G | 742.2439878 | 0.018403 |
| w ~ 1 + D + T + G + D:G | 742.2439878 | 0.018403 |
| w ~ 1 + D + G:T | 744.1016085 | 0.00727 |
| w ~ 1 + D + T + G:T | 744.1016085 | 0.00727 |
| w ~ 1 + T + G + D:T | 744.1660856 | 0.007039 |
| w ~ 1 + D + G | 744.5755343 | 0.005736 |
| w ~ 1 + T + G | 744.6397694 | 0.005555 |
| w ~ 1 + D + T + G + D:T | 745.1157629 | 0.004378 |

**Table S3.** ANOVA of predictors for the best fit-model. Significance levels: ***p < 0.001, **p < 0.01, *p < 0.05. Df – degree of freedom, Pr(>Chi) – p-value associated with the Chi²-test. For categories see Table S3.

| Term | Df | Deviance | Residual Df | Residual Deviance | Pr(>Chi) |
| --- | --- | --- | --- | --- | --- |
| NULL |  |  | 70 | 197.92 |  |
| Divergence time | 1 | 19.351 | 69 | 178.57 | 0.0002906*** |
| Dispersal category | 6 | 54.883 | 63 | 123.69 | 1.582e-06*** |
| Taxonomic Group | 4 | 23.183 | 59 | 100.51 | 0.0034038** |

**Table S4.** Predictors and regression values for the best-fit model. Predictor interactions without occurrences have been omitted for clarity. N, number of occurrences. ^1^*p<0.05; **p<0.01; ***p<0.001, ^2^CI = confidence interval, ^3^False discovery rate correction for multiple testing.

| Predictor | N | exp(Beta)^1^ | 95% CI^2^ | p-value | q-value^3^ |
| --- | --- | --- | --- | --- | --- |
| Divergence time estimates | 71 | 0.78*** | 0.67, 0.90 | <0.001 | 0.006 |
| Group |  |  |  |  |  |
| Lissamphibia (reference) | 25 | — | — |  |  |
| Aves | 19 | 0.97 | 0.33, 3.16 | >0.9 | >0.9 |
| Hexapoda | 5 | 0.13** | 0.03, 0.61 | 0.003 | 0.019 |
| Mammalia | 8 | 0.16** | 0.05, 0.58 | 0.007 | 0.020 |
| Squamata | 14 | 1.21 | 0.41, 3.62 | 0.7 | 0.8 |
| Dispersal category |  |  |  |  |  |
| C (reference) | 21 | — | — |  |  |
| D | 18 | 3.52** | 1.26, 10.2 | 0.006 | 0.020 |
| A | 13 | 0.79 | 0.31, 2.08 | 0.7 | 0.8 |
| B | 7 | 0.23* | 0.08, 0.80 | 0.015 | 0.034 |
| E | 6 | 4.39* | 1.11, 19.7 | 0.031 | 0.056 |
| G | 5 | 0.68 | 0.18, 3.09 | 0.6 | 0.8 |
| H | 1 | 0.62 | 0.08, 29.8 | 0.7 | 0.8 |

**Table S5.** Descriptive statistics of divergence time in million years (Ma) for species pairs obtained from the analysis of seven timetrees from timetree.org.

| Taxonomic group | n | mean divergence time (Ma) | median divergence time (Ma) | standard deviation of divergence time (Ma) |
| --- | --- | --- | --- | --- |
| Aves | 4415 | 6.19 | 3.93 | 7.55 |
| Squamata | 3989 | 16.2 | 12.8 | 13.5 |
| Mammalia | 2887 | 6.82 | 4.77 | 6.94 |
| Lissamphibia | 2523 | 14.3 | 10.7 | 13.3 |
| Neoteleostei | 5419 | 13.4 | 8.65 | 14.1 |
| Lepidoptera | 2109 | 15.9 | 10.1 | 20.5 |
| Mollusca | 1565 | 35.5 | 13.6 | 60.8 |

Supplementary References

Aldrich, B.T., and Kambhampati, S. 2009. Preliminary analysis of a hybrid zone between two subspecies of *Zootermopsis nevadensis*. Insectes Sociaux, 56(4), 439–450.

Alexandrino, J., Baird, S.J.E., Lawson, L., Macey, J.R., Moritz, C., and Wake, D.B. 2005. Strong selection against hybrids at a hybrid zone in the *Ensatina* ring species complex and its evolutionary implications. Evolution, 59(6), 1334–1347.

Alexandrino, J., Froufe, E., Arntzen, J.W., and Ferrand, N. 2000. Genetic subdivision, glacial refugia and postglacial recolonization in the golden-striped salamander, *Chioglossa lusitanica* (Amphibia: Urodela). Molecular Ecology, 9(6), 771–781

Arntzen, J.W. 1980. Ecological observations on *Chioglossa lusitanica* (Caudata, Salamandridae). Amphibia-Reptilia, 3(4), 187–203.

Arntzen, J.W. 2018. Morphological and molecular characters to describe a marbled newt hybrid zone in the Iberian Peninsula. Contributions to Zoology, 87(3), 167–185.

Arntzen, J.W., de Vries, W., Canestrelli, D., and Martínez-Solano, I. 2017. Hybrid zone formation and contrasting outcomes of secondary contact over transects in common toads. Molecular Ecology, 26(20), 5663–5675.

Arntzen, J.W., Espregueira Themudo, G., and Wielstra, B. 2007. The phylogeny of crested newts (Triturus cristatus superspecies): nuclear and mitochondrial genetic characters suggest a hard polytomy, in line with the paleogeography of the centre of origin. Contributions to Zoology, 76(4), 261–278.

Arntzen, J.W., Trujillo, T., Butôt, R., Vrieling, K., Schaap, O., Gutiérrez-Rodríguez, J., and Martínez-Solano, I. 2016. Concordant morphological and molecular clines in a contact zone of the Common and Spined toad (*Bufo bufo* and *B. spinosus*) in the northwest of France. Frontiers in Zoology, 13(1), 52.

Asztalos, M., Schultze, N., Ihlow, F., Geniez, P., Berroneau, M., Delmas, C., Guiller, G., Legentilhomme, J., Kindler, C., and Fritz, U. 2020. How often do they do it? An in-depth analysis of the hybrid zone of two grass snake species (*Natrix astreptophora* and *Natrix helvetica*). Biological Journal of the Linnean Society, 131(4), 756–773.

Baldassarre, D.T., White, T.A., Karubian, J., and Webster, M.S. 2014. Genomic and morphological analysis of a semipermeable avian hybrid zone suggests asymmetrical introgression of a sexual signal. Evolution, 68(9), 2644–2657.

Barrowclough, G.F., Groth, J.G., Mertz, L.A., and Gutiérrez, R.J. 2005. Genetic structure, introgression, and a narrow hybrid zone between northern and California spotted owls (*Strix occidentalis*). Molecular Ecology, 14(4), 1109–1120.

Batalha-Filho, H., Maldonado-Coelho, M., and Miyaki, C.Y. 2019. Historical climate changes and hybridization shaped the evolution of Atlantic Forest spinetails (Aves: Furnariidae). Heredity, 123(5), 675–693.

Bernard-Laurent, A. 1991. Migrant rock partridges (*Alectoris graeca saxatilis*) in the southern French Alps. Journal für Ornithologie, 132(2), 220–223.

Bert, T.M., and Harrison, R.G. 1988. Hybridization in western Atlantic stone crabs (genus *Menippe*): evolutionary history and ecological context influence species interactions. Evolution, 42(3), 528–544.

Berven, K.A., and Grudzien, T.A. 1990. Dispersal in the wood frog (*Rana sylvatica*): implications for genetic population structure. Evolution, 44(8), 2047–2056.

Beysard, M., and Heckel, G. 2014. Structure and dynamics of hybrid zones at different stages of speciation in the common vole (*Microtus arvalis*). Molecular Ecology, 23(3), 673–687.

Binzenhöfer, B., Schröder, B., Strauss, B., Biedermann, R., and Settele, J. 2005. Habitat models and habitat connectivity analysis for butterflies and burnet moths – the example of *Zygaena carniolica* and *Coenonympha arcania*. Biological Conservation, 126(2), 247–259.

Božíková, E., Munclinger, P., Teeter, K.C., Tucker, P.K., Macholán, M., and Piálek, J. 2005. Mitochondrial DNA in the hybrid zone between *Mus musculus musculus* and *Mus musculus domesticus*: a comparison of two transects. Biological Journal of the Linnean Society, 84(3), 363–378.

Brelsford, A., and Irwin, D.E. 2009. Incipient speciation despite little assortative mating: the yellow-rumped warbler hybrid zone. Evolution, 63(12), 3050–3060.

Brumfield, R.T. 2005. Mitochondrial variation in Bolivian populations of the variable antshrike (*Thamnophilus caerulescens*). The Auk, 122(2), 414–432.

Burbrink, F.T., Gehara, M., McKelvy, A.D., and Myers, E.A. 2021. Resolving spatial complexities of hybridization in the context of the gray zone of speciation in North American ratsnakes (*Pantherophis obsoletus* complex). Evolution, 75(2), 260–277.

Caeiro-Dias, G., Rocha, S., Couto, A., Pereira, C., Brelsford, A., Crochet, P.-A., and Pinho, C. 2021. Nuclear phylogenies and genomics of a contact zone establish the species rank of *Podarcis lusitanicus* (Squamata, Lacertidae). Molecular Phylogenetics and Evolution, 164, 107270.

Calsbeek, R., Duryea, M.C., Parker, E., and Cox, R.M. 2014. Sex-biased juvenile dispersal is adaptive but does not create genetic structure in island lizards. Behavioral Ecology, 25(5), 1157–1163.

Capblancq, T., Després, L., and Mavárez, J. 2020. Genetic, morphological and ecological variation across a sharp hybrid zone between two alpine butterfly species. Evolutionary Applications, 13(6), 1435–1450.

Carling, M.D., and Brumfield, R.T. 2008. Haldane’s rule in an avian system: using cline theory and divergence population genetics to test for differential introgression of mitochondrial, autosomal, and sex-linked loci across the *Passerina* bunting hybrid zone. Evolution, 62(10), 2600–2615.

Carling, M.D., and Zuckerberg, B. 2011. Spatio‐temporal changes in the genetic structure of the *Passerina* bunting hybrid zone. Molecular Ecology, 20(6), 1166–1175.

Carling, M.D., Serene, L.G., and Lovette, I.J. 2011. Using historical DNA to characterize hybridization between Baltimore orioles (*Icterus galbula*) and Bullock’s orioles (*I. bullockii*). The Auk, 128(1), 61–68.

Carmen, W.J. 1988. Behavioral ecology of the California scrub jay (Aphelocoma coerulescens californica): a non-cooperative breeder with close cooperative relatives. Doctoral dissertation, University of California, Berkeley, USA

Carneiro, M., Baird, S.J.E., Afonso, S., Ramirez, E., Tarroso, P., Teotónio, H., Villafuerte, R., Nachman, M.W., and Ferrand, N. 2013. Steep clines within a highly permeable genome across a hybrid zone between two subspecies of the European rabbit. Molecular Ecology, 22(9), 2511–2525.

Carneiro, M., Baird, S.J.E., Afonso, S., Ramirez, E., Tarroso, P., Teotónio, H., Villafuerte, R., Nachman, M.W., and Ferrand, N. 2013. Steep clines within a highly permeable genome across a hybrid zone between two subspecies of the European rabbit. Molecular Ecology, 22(9), 2511–2525.

Case, S.M., and Williams, E.E. 1984. Study of a contact zone in the *Anolis distichus* complex in the Central Dominican Republic. Herpetologica, 40(2), 118–137.

Chavez, A.S., Maher, S.P., Arbogast, B.S., and Kenagy, G.J. 2014. Diversification and gene flow in nascent lineages of island and mainland north American tree squirrels (*Tamiasciurus*). Evolution, 68(4), 1094–1109.

Chavez, A.S., Saltzberg, C.J., and Kenagy, G.J. 2011. Genetic and phenotypic variation across a hybrid zone between ecologically divergent tree squirrels (*Tamiasciurus*). Molecular Ecology, 20(16), 3350–3366.

Cheng, J., Czypionka, T., and Nolte, A.W. 2013. The genomics of incompatibility factors and sex determination in hybridizing species of *Cottus* (Pisces). Heredity, 111(6), 520–529.

Conroy, C.J., and Gupta, A.M. 2011. Cranial morphology of the California vole (*Microtus californicus*, Cricetidae) in a contact zone. Biological Journal of the Linnean Society, 104(2), 264–283.

Cronemberger, Á.A., Aleixo, A., Mikkelsen, E.K., and Weir, J.T. 2020. Postzygotic isolation drives genomic speciation between highly cryptic *Hypocnemis* antbirds from Amazonia. Evolution, 74(11), 2512–2525.

Dasmahapatra, K.K., Blum, M.J., Aiello, A., Hackwell, S., Davies, N., Bermingham, E.P., and Mallet, J. 2002. Inferences from a rapidly moving hybrid zone. Evolution, 56(4), 741–753.

Delmore, K.E., Brenneman, R.A., Lei, R., Bailey, C.A., Brelsford, A., Louis, E.E., and Johnson, S.E. 2013. Clinal variation in a brown lemur (*Eulemur* spp.) hybrid zone: combining morphological, genetic and climatic data to examine stability. Journal of Evolutionary Biology, 26(8), 1677–1690.

Del‐Rio, G., Rego, M.A., Whitney, B.M., Schunck, F., Silveira, L.F., Faircloth, B.C., and Brumfield, R.T. 2021. Displaced clines in an avian hybrid zone (Thamnophilidae: *Rhegmatorhina*) within an Amazonian interfluve. Evolution, evo.14377.

Dessauer, H.C., Cole, C.J., and Townsend, C.R. 2000. Hybridization among western whiptail lizards (*Cnemidophorus tigris*) in southwestern New Mexico: Population genetics, morphology, and ecology in three contact zones. Bulletin of the American Museum of Natural History, 246, 1–148.

Devitt, T.J., Baird, S.J., and Moritz, C. 2011. Asymmetric reproductive isolation between terminal forms of the salamander ring species *Ensatina eschscholtzii* revealed by fine-scale genetic analysis of a hybrid zone. BMC Evolutionary Biology, 11(245), 1–14.

Dod, B., Smadja, C., Karn, R.C., and Boursot, P. 2005. Testing for selection on the androgen-binding protein in the Danish mouse hybrid zone. Biological Journal of the Linnean Society, 84(3), 447–459.

Dufková, P., Macholán, M., and Piálek, J. 2011. Inference of selection and stochastic effects in the house mouse hybrid zone. Evolution, 65(4), 993–1010.

Dufresnes, C., and Martínez-Solano, Í. 2020. Hybrid zone genomics supports candidate species in Iberian *Alytes obstetricans.* Amphibia-Reptilia, 41(1), 105–112.

Dufresnes, C., Berroneau, M., Dubey, S., Litvinchuk, S.N., and Perrin, N. 2020c. The effect of phylogeographic history on species boundaries: a comparative framework in *Hyla* tree frogs. Scientific Reports, 10(1), 5502.

Dufresnes, C., Bonato, L., Novarini, N., Betto-Colliard, C., Perrin, N., and Stöck, M. 2014. Inferring the degree of incipient speciation in secondary contact zones of closely related lineages of Palearctic green toads (*Bufo viridis* subgroup). Heredity, 113(1), 9–20.

Dufresnes, C., Brelsford, A., Crnobrnja-Isailović, J., Tzankov, N., Lymberakis, P., and Perrin, N. 2015. Timeframe of speciation inferred from secondary contact zones in the european tree frog radiation (*Hyla arborea* group). BMC Evolutionary Biology, 15(1), 155.

Dufresnes, C., Brelsford, A., Jeffries, D.L., Mazepa, G., Suchan, T., Canestrelli, D., Nicieza, A., Fumagalli, L., Dubey, S., Martínez-Solano, I., Litvinchuk, S.N., Vences, M., Perrin, N., and Crochet, P.-A. 2021a. Mass of genes rather than master genes underlie the genomic architecture of amphibian speciation. Proceedings of the National Academy of Sciences, 118(36), e2103963118.

Dufresnes, C., Litvinchuk, S.N., Rozenblut-Kościsty, B., Rodrigues, N., Perrin, N., Crochet, P.-A., and Jeffries, D.L. 2020d. Hybridization and introgression between toads with different sex chromosome systems. Evolution Letters, 4(5), 444–456.

Dufresnes, C., Mazepa, G., Rodrigues, N., Brelsford, A., Litvinchuk, S.N., Sermier, R., Lavanchy, G., Betto-Colliard, C., Blaser, O., Borzée, A., Cavoto, E., Fabre, G., Ghali, K., Grossen, C., Horn, A., Leuenberger, J., Phillips, B.C., Saunders, P.A., Savary, R., Maddalena, T., Stöck, M., Dubey, S., Canestrelli, D., and Jeffries, D.L. 2018. Genomic evidence for cryptic speciation in tree frogs from the Apennine Peninsula, with description of *Hyla perrini* sp. nov. Frontiers in Ecology and Evolution, 6.

Dufresnes, C., Nicieza, A.G., Litvinchuk, S.N., Rodrigues, N., Jeffries, D.L., Vences, M., Perrin, N., and Martínez‐Solano, Í. 2020b. Are glacial refugia hotspots of speciation and cytonuclear discordances? Answers from the genomic phylogeography of Spanish common frogs. Molecular Ecology, 29(5), 986–1000.

Dufresnes, C., Pribille, M., Alard, B., Gonçalves, H., Amat, F., Crochet, P.-A., Dubey, S., Perrin, N., Fumagalli, L., Vences, M., and Martínez-Solano, I. 2020a. Integrating hybrid zone analyses in species delimitation: lessons from two anuran radiations of the Western Mediterranean. Heredity, 124(3), 423–438.

Dufresnes, C., Strachinis, I., Tzoras, E., Litvinchuk, S.N., and Denoël, M. 2019. Call a spade a spade: taxonomy and distribution of *Pelobates*, with description of a new Balkan endemic. ZooKeys, 859, 131–158.

Dufresnes, C., Suchan, T., Smirnov, N.A., Denoël, M., Rosanov, J.M., and Litvinchuk, S.N. 2021b. Revisiting a speciation classic: comparative analyses support sharp but leaky transitions between *Bombina* toads. Journal of Biogeography, 48(3), 548–560.

Ebdon, S., Laetsch, D.R., Dapporto, L., Hayward, A., Ritchie, M.G., Dincӑ, V., Vila, R., and Lohse, K. 2021. The Pleistocene species pump past its prime: evidence from European butterfly sister species. Molecular Ecology, 30(14), 3575–3589.

Ehl, S., Vences, M., and Veith, M. 2019. Reconstructing evolution at the community level: A case study on Mediterranean amphibians. Molecular Phylogenetics and Evolution, 134, 211–225.

Ehrhardt, N.M., Die, D.J., and Restrepo, V.R. 1990. Abundance and impact of fishing on a stone crab (*Menippe mercenerla*) population in Everglades National Park, Florida. Bulletin of Marine Science, 46(2), 311–323.

Engebretsen, K.N., Barrow, L.N., Rittmeyer, E.N., Brown, J.M., and Moriarty Lemmon, E. 2016. Quantifying the spatiotemporal dynamics in a chorus frog (*Pseudacris*) hybrid zone over 30 years. Ecology and Evolution, 6(14), 5013–5031.

Erickson, R.A., Hamilton, R.A., and Mlodinow, S.G. 2008. Status review of Belding’s yellowthroat *Geothlypis beldingi*, and implications for its conservation. Bird Conservation International, 18(3), 219–228.

Fritz, U., Corti, C., and Päckert, M. 2012. Mitochondrial DNA sequences suggest unexpected phylogenetic position of Corso-Sardinian grass snakes (*Natrix cetti*) and do not support their species status, with notes on phylogeography and subspecies delineation of grass snakes. Organisms Diversity & Evolution, 12(1), 71–80.

García-Porta, J., Litvinchuk, S.N., Crochet, P.A., Romano, A., Geniez, P.H., Lo-Valvo, M., Lymberakis, P., and Carranza, S. 2012. Molecular phylogenetics and historical biogeography of the West-Palearctic common toads (*Bufo bufo* species complex). Molecular Phylogenetics and Evolution, 63(1), 113–130.

Geneva, A.J., Hilton, J., Noll, S., and Glor, R.E. 2015. Multilocus phylogenetic analyses of Hispaniolan and Bahamian trunk anoles (*Distichus* species group). Molecular Phylogenetics and Evolution, 87, 105–117.

Gifford, M.E. 2008. Divergent character clines across a recent secondary contact zone in a Hispaniolan lizard. Journal of Zoology, 274(3), 292–300.

Gifford, M.E., and Larson, A. 2008. In situ genetic differentiation in a Hispaniolan lizard (*Ameiva chrysolaema*): a multilocus perspective. Molecular Phylogenetics and Evolution, 49(1), 277–291.

Gilg, M.R., and Hilbish, T.J. 2003. The geography of marine larval dispersal: coupling genetics with fine-scale physical oceanography. Ecology, 84(11), 2989–2998.

Gjerdrum, C., Elphick, C.S., and Rubega, M.A. 2008. How well can we model numbers and productivity of saltmarsh sharp-tailed sparrows (*Ammodramus caudacutus*) using habitat features? The Auk, 125(3), 608–617.

Gonçalves, H., Maia-Carvalho, B., Sousa-Neves, T., García-París, M., Sequeira, F., Ferrand, N., and Martínez-Solano, I. 2015. Multilocus phylogeography of the common midwife toad, *Alytes obstetricans* (Anura, Alytidae): contrasting patterns of lineage diversification and genetic structure in the Iberian refugium. Molecular Phylogenetics and Evolution, 93, 363–379.

González‐Villaseñor, L.I., and Powers, D.A. 1990. Mitochondrial‐DNA restriction‐site polymorphisms in the Teleost *Fundulus heteroclitus* support secondary intergradation. Evolution, 44(1), 27–37.

Gowen, F.C., Maley, J.M., Cicero, C., Peterson, A.T., Faircloth, B.C., Warr, T.C., and McCormack, J.E. 2014. Speciation in western scrub-jays, Haldane’s rule, and genetic clines in secondary contact. BMC Evolutionary Biology, 14(1), 135.

Grossen, C., Seneviratne, S.S., Croll, D., and Irwin, D.E. 2016. Strong reproductive isolation and narrow genomic tracts of differentiation among three woodpecker species in secondary contact. Molecular Ecology, 25(17), 4247–4266.

Haig, S.M., Mullins, T.D., and Forsman, E.D. 2004. Subspecific relationships and genetic structure in the spotted owl. Conservation Genetics, 5(5), 683–705.

Hare, M.P., and Avise, J.C. 1996. Molecular genetic analysis of a stepped multilocus cline in the American oyster (*Crassostrea virginica*). Evolution, 50(6), 2305–2315.

Heckel, G., Burri, R., Fink, S., Desmet, J.-F., and Excoffier, L. 2005. Genetic structure and colonization processes in European populations of the common vole, *Microtus arvalis.* Evolution, 59(10), 2231–2242.

Hill, R.I., Gilbert, L.E., and Kronforst, M.R. 2013. Cryptic genetic and wing pattern diversity in a mimetic *Heliconius* butterfly. Molecular Ecology, 22(10), 2760–2770.

Hofman, S., and Szymura, J.M. 2007. Limited mitochondrial DNA introgression in a *Bombina* hybrid zone. Biological Journal of the Linnean Society, 91(2), 295–306.

Horníková, M., Marková, S., Lanier, H.C., Searle, J.B., and Kotlík, P. 2021. A dynamic history of admixture from Mediterranean and Carpathian glacial refugia drives genomic diversity in the bank vole. Ecology and Evolution, 11(12), 8215–8225.

Irwin, D.E. 2002. Phylogeographic breaks without geographic barriers to gene flow. Evolution, 56(12), 2383–2394.

Jackson, J.D., Zefania, S., Moehy, S., Bamford, A.J., Bruford, M.W., and Székely, T. 2019. Ecology, conservation, and phylogenetic position of the Madagascar jacana *Actophilornis albinucha.* Ostrich, 90(4), 315–326.

Jacobsen, F., and Omland, K.E. 2012. Extensive introgressive hybridization within the northern oriole group (genus *Icterus*) revealed by three‐species isolation with migration analysis. Ecology and Evolution, 2(10), 2413–2429.

Johansson, H., Surget‐Groba, Y., and Thorpe, R.S. 2008. The roles of allopatric divergence and natural selection in quantitative trait variation across a secondary contact zone in the lizard *Anolis roquet*. Molecular Ecology, 17(23), 5146–5156.

Kawakami, T., Butlin, R.K., Adams, M., Paull, David.J., and Cooper, S.J.B. 2009. Genetic analysis of a chromosomal hybrid zone in the Australian morabine grasshoppers (*Vandiemenella*, *Viatica* species group). Evolution, 63(1), 139–152.

Kawakami, T., Butlin, R.K., Adams, M., Saint, K.M., Paull, D.J., and Cooper, S.J.B. 2007. Differential gene flow of mitochondrial and nuclear DNA markers among chromosomal races of Australian morabine grasshoppers (*Vandiemenella, viatica* species group). Molecular Ecology, 16(23), 5044–5056.

Kindler, C., Chèvre, M., Ursenbacher, S., Böhme, W., Hille, A., Jablonski, D., Vamberger, M., and Fritz, U. 2017. Hybridization patterns in two contact zones of grass snakes reveal a new Central European snake species. Scientific Reports, 7(1), 7378.

Kindler, C., De Pous, P., Carranza, S., Beddek, M., Geniez, P., and Fritz, U. 2018. Phylogeography of the Ibero-Maghrebian red-eyed grass snake (*Natrix astreptophora*). Organisms Diversity & Evolution, 18(1), 143–150.

Kozak, K.M., Wahlberg, N., Neild, A.F.E., Dasmahapatra, K.K., Mallet, J., and Jiggins, C.D. 2015. Multilocus species trees show the recent adaptive radiation of the mimetic *Heliconius* butterflies. Systematic Biology, 64(3), 505–524.

Kozakiewicz, M., Chołuj, A., and Kozakiewicz, A. 2007. Long-distance movements of individuals in a free-living bank vole population: an important element of male breeding strategy. Acta Theriologica, 52(4), 339–348.

Kuchta, S.R. 2007. Contact zones and species limits: hybridization between lineages of the California newt, *Taricha torosa*, in the southern Sierra Nevada. Herpetologica, 63(3), 332–350.

Kuchta, S.R., and Tan, A.-M. 2006. Lineage diversification on an evolving landscape: phylogeography of the California newt, *Taricha torosa* (Caudata: Salamandridae). Biological Journal of the Linnean Society, 89(2), 213–239.

Kuchta, S.R., Parks, D.S., Mueller, R.L., and Wake, D.B. 2009. Closing the ring: historical biogeography of the salamander ring species *Ensatina eschscholtzii*. Journal of Biogeography, 36(5), 982–995.

Kunte, K., Shea, C., Aardema, M.L., Scriber, J.M., Juenger, T.E., Gilbert, L.E., and Kronforst, M.R. 2011. Sex chromosome mosaicism and hybrid speciation among tiger swallowtail butterflies. PLoS Genetics, 7(9), e1002274.

Larson, E.L., White, T.A., Ross, C.L., and Harrison, R.G. 2014. Gene flow and the maintenance of species boundaries. Molecular Ecology, 23(7), 1668–1678.

Leaché, A.D., and Cole, C.J. 2007. Hybridization between multiple fence lizard lineages in an ecotone: locally discordant variation in mitochondrial DNA, chromosomes, and morphology. Molecular Ecology, 16(5), 1035–1054.

Leaché, A.D., Banbury, B.L., Linkem, C.W., and De Oca, A.N.-M. 2016. Phylogenomics of a rapid radiation: is chromosomal evolution linked to increased diversification in North American spiny lizards (genus *Sceloporus*)? BMC Evolutionary Biology, 16(1), 63.

Leaché, A.D., Grummer, J.A., Harris, R.B., and Breckheimer, I.K. 2017. Evidence for concerted movement of nuclear and mitochondrial clines in a lizard hybrid zone. Molecular Ecology, 26(8), 2306–2316.

Lederhouse, R.C. 1983. Population structure, residency and weather related mortality in the black swallowtail butterfly, *Papilio polyxenes*. Oecologia, 59(2–3), 307–311.

Lee, J.Y., and Edwards, S.V. 2008. Divergence across Australia’s Carpentarian barrier: statistical phylogeography of the red-backed fairy wren (*Malurus melanocephalus*). Evolution, 62(12), 3117–3134.

Lemmon, A.R., and Lemmon, E.M. 2008. A likelihood framework for estimating phylogeographic history on a continuous landscape. Systematic Biology, 57(4), 544–561.

Lemmon, E.M., Lemmon, A.R., and Cannatella, D.C. 2007. Geological and climatic forces driving speciation in the continentally distributed trilling chorus frogs (*Pseudacris*). Evolution, 61(9), 2086–2103.

Lidicker, W.Z.Jr., and Patton, J.L. 1987. Patterns of dispersal and genetic structure in populations of small rodents. In, Mammalian dispersal patterns: the effects of social structure on population genetics, (B. D. Chepko-Sade and Z. T. Halpin, Editors). The University of Chicago Press, Chicago, USA.

Lin, D., Bi, K., Conroy, C.J., Lacey, E.A., Schraiber, J.G., and Bowie, R.C.K. 2018. Mito-nuclear discordance across a recent contact zone for California voles. Ecology and Evolution, 8(12), 6226–6241.

Linck, E., Epperly, K., Van Els, P., Spellman, G.M., Bryson, R.W., McCormack, J.E., Canales-Del-Castillo, R., and Klicka, J. 2019. Dense geographic and genomic sampling reveals paraphyly and a cryptic lineage in a classic sibling species complex. Systematic Biology, 956–966.

Lipshutz, S.E., Meier, J.I., Derryberry, G.E., Miller, M.J., Seehausen, O., and Derryberry, E.P. 2019. Differential introgression of a female competitive trait in a hybrid zone between sex‐role reversed species. Evolution, 73(2), 188–201.

Lopez, K.A., McDiarmid, C.S., Griffith, S.C., Lovette, I.J., and Hooper, D.M. 2021. Evaluating evidence of mitonuclear incompatibilities with the sex chromosomes in an avian hybrid zone. Evolution, 75(6), 1395–1414.

Lovell, S.F., Lein, M.R., and Rogers, S.M. 2021. Cryptic speciation in the Warbling Vireo (Vireo gilvus). Ornithology, 138(1), 1–16.

Lunt, D.H., Ibrahim, K.M., and Hewitt, G.M. 1998. mtDNA phylogeography and postglacial patterns of subdivision in the meadow grasshopper *Chorthippus parallelus*. Heredity, 80, 633–641.

Macholán, M., Munclinger, P., Šugerková, M., Dufková, P., Bímová, B., Božíková, E., Zima, J., and Piálek, J. 2007. Genetic analysis of autosomal and X-linked markers across a mouse hybrid zone. Evolution, 61(4), 746–771.

Mallet, J., Barton, N., Lamas, G., Santisteban, J., Muedas, M., and Eeley, H. 1990. Estimates of selection and gene flow from measures of cline width and linkage disequilibrium in *Heliconius* hybrid zones. Genetics, 124(4), 921–936.

Markolf, M., and Kappeler, P.M. 2013. Phylogeographic analysis of the true lemurs (genus *Eulemur*) underlines the role of river catchments for the evolution of micro-endemism in Madagascar. Frontiers in Zoology, 10(1), 70.

Maroja, L.S., Andrés, J.A., and Harrison, R.G. 2009. Genealogical discordance and patterns of introgression and selection across a cricket hybrid zone. Evolution, 63(11), 2999–3015.

Massot, M. 2003. Genetic, prenatal, and postnatal correlates of dispersal in hatchling fence lizards (*Sceloporus occidentalis*). Behavioral Ecology, 14(5), 650–655.

Mathewson, H.A., Morrison, M.L., Loffland, H.L., and Brussard, P.F. 2013. Ecology of willow flycatchers (*Empidonax traillii*) in the Sierra Nevada, California: effects of meadow characteristics and weather on demographics. Ornithological Monographs, 75(1), 1–32.

McCormack, J.E., Heled, J., Delaney, K.S., Peterson, A.T., and Knowles, L.L. 2011. Calibrating divergence times on species trees versus gene trees: implications for speciation history of *Aphelocoma* jays. Evolution, 65(1), 184–202.

McKenzie, J.L., Dhillon, R.S., and Schulte, P.M. 2015. Evidence for a bimodal distribution of hybrid indices in a hybrid zone with high admixture. Royal Society Open Science, 2(12), 150285.

McKenzie, J.L., Dhillon, R.S., and Schulte, P.M. 2016. Steep, coincident, and concordant clines in mitochondrial and nuclear‐encoded genes in a hybrid zone between subspecies of Atlantic killifish, *Fundulus heteroclitus*. Ecology and Evolution, 6(16), 5771–5787.

Mettler, R.D., and Spellman, G.M. 2009. A hybrid zone revisited: molecular and morphological analysis of the maintenance, movement, and evolution of a Great Plains avian (Cardinalidae: *Pheucticus*) hybrid zone. Molecular Ecology, 18(15), 3256–3267.

Milá, B., Toews, D.P.L., Smith, T.B., and Wayne, R.K. 2011. A cryptic contact zone between divergent mitochondrial DNA lineages in southwestern North America supports past introgressive hybridization in the yellow-rumped warbler complex (Aves: *Dendroica coronata*). Biological Journal of the Linnean Society, 103(3), 696–706.

Miller, M.J., Lipshutz, S.E., Smith, N.G., and Bermingham, E. 2014. Genetic and phenotypic characterization of a hybrid zone between polyandrous northern and wattled jacanas in western Panama. BMC Evolutionary Biology, 14(1), 227.

Miraldo, A., Faria, C., Hewitt, G.M., Paulo, O.S., and Emerson, B.C. 2013. Genetic analysis of a contact zone between two lineages of the ocellated lizard (*Lacerta lepida* Daudin 1802) in south-eastern Iberia reveal a steep and narrow hybrid zone. Journal of Zoological Systematics and Evolutionary Research, 51(1), 45–54.

Moore, W.S., and Buchanan, E.B. 1985. Stability of the northern flicker hybrid zone in historical times: implications for adaptive speciation theory. Evolution, 39(1), 135–151.

Moore, W.S., and Dolbeer, R.A. 1989. The use of banding recovery data to estimate dispersal rates and gene flow in avian species: case studies in the red-winged blackbird and common grackle. The Condor, 91(2), 242.

Morgan-Richards, M., and Wallis, G.P. 2003. A comparison of five hybrid zones of the weta *Hemideina thoracica* (Orthoptera: Anostostomatidae): degree of cytogenetic differentiation fails to predict zone width. Evolution, 57(4), 849–861.

Morgan-Richards, M., Trewick, S.A., and Wallis, G.P. 2000. Characterization of a hybrid zone between two chromosomal races of the weta *Hemideina thoracica* following a geologically recent volcanic eruption. Heredity, 85(6), 586–592.

Moyle, R.G., Filardi, C.E., Smith, C.E., and Diamond, J. 2009. Explosive Pleistocene diversification and hemispheric expansion of a “great speciator.” Proceedings of the National Academy of Sciences, 106(6), 1863–1868.

Nolte, A.W., Freyhof, J., and Tautz, D. 2006. When invaders meet locally adapted types: rapid moulding of hybrid zones between sculpins (*Cottus*, Pisces) in the Rhine system. Molecular Ecology, 15(7), 1983–1993.

Nolte, A.W., Freyhof, J., Stemshorn, K.C., and Tautz, D. 2005. An invasive lineage of sculpins, *Cottus* sp. (Pisces, Teleostei) in the Rhine with new habitat adaptations has originated from hybridization between old phylogeographic groups. Proceedings of the Royal Society B: Biological Sciences, 272(1579), 2379–2387.

Oatley G., Voelker G., Crowe T.M., Bowie R.C.K. 2012. A multi-locus phylogeny reveals a complex pattern of diversification related to climate and habitat heterogeneity in southern African white-eyes. Molecular Phylogenetics and Evolution. 64:633–644.

Oatley, G., De Swardt, D.H., Nuttall, R.J., Crowe, T.M., and Bowie, R.C.K. 2017. Phenotypic and genotypic variation across a stable white-eye (*Zosterops* sp.) hybrid zone in central South Africa. Biological Journal of the Linnean Society, 121(3), 670–684.

Osorio-Beristain, M., and Drummond, H. 1993. Natal dispersal and deferred breeding in the blue-footed booby. The Auk, 110(2), 234–239.

Pabijan, M., Wandycz, A., Hofman, S., Węcek, K., Piwczyński, M., and Szymura, J.M. 2013. Complete mitochondrial genomes resolve phylogenetic relationships within *Bombina* (Anura: Bombinatoridae). Molecular Phylogenetics and Evolution, 69(1), 63–74.

Paradis, E., Baillie, S.R., Sutherland, W.J., and Gregory, R.D. 1998. Patterns of natal and breeding dispersal in birds. Journal of Animal Ecology, 67(4), 518–536.

Patterson, S.A., Morris-Pocock, J.A., and Friesen, V.L. 2011. A multilocus phylogeny of the Sulidae (Aves: Pelecaniformes). Molecular Phylogenetics and Evolution, 58(2), 181–191.

Payseur, B.A., Krenz, J.G., and Nachman, M.W. 2004. Differential patterns of introgression across the X chromosome in a hybrid zone between two species of house mice. Evolution, 58(9), 2064–2078.

Phillips, B.L., Baird, S.J.E., and Moritz, C. 2004. When vicars meet: a narrow contact zone between morphologically cryptic phylogeographic lineages of the rainforest skink, *Carlia rubrigularis*. Evolution, 58(7), 1536–1548.

Polyakov, A.V., White, T.A., Jones, R.M., Borodin, P.M., and Searle, J.B. 2011. Natural hybridization between extremely divergent chromosomal races of the common shrew (*Sorex araneus*, Soricidae, Soricomorpha): hybrid zone in Siberia. Journal of Evolutionary Biology, 24(7), 1393–1402.

Porter, A.H., Wenger, R., Geiger, H., Scholl, A., and Shapiro, A.M. 1997. The Pontia daplidice-edusa hybrid zone in northwestern Italy. Evolution, 51(5), 1561–1573.

Pulgarín-R, P.C., Smith, B.T., Bryson, R.W., Spellman, G.M., and Klicka, J. 2013. Multilocus phylogeny and biogeography of the New World *Pheucticus* grosbeaks (Aves: Cardinalidae). Molecular Phylogenetics and Evolution, 69(3), 1222–1227.

Pulido-Santacruz, P., Aleixo, A., and Weir, J.T. 2018. Morphologically cryptic Amazonian bird species pairs exhibit strong postzygotic reproductive isolation. Proceedings of the Royal Society B: Biological Sciences, 285(1874), 20172081.

Qi, Y., Lu, B., Gao, H., Hu, P., and Fu, J. 2014. Hybridization and mitochondrial genome introgression between *Rana chensinensis* and *R. kukunoris*. Molecular Ecology, 23(22), 5575–5588.

Randi, E., and Bernard-Laurent, A. 1999. Population genetics of a hybrid zone between the red-legged partridge and rock partridge. The Auk, 116(2), 324–337.

Raufaste, N., Orth, A., Belkhir, K., Senet, D., Smadja, C., Baird, S.J.E., Bonhomme, F., Dod, B., and Boursot, P. 2005. Inferences of selection and migration in the Danish house mouse hybrid zone. Biological Journal of the Linnean Society, 84(3), 593–616.

Reeb, C.A., and Avise, J.C. 1990. A genetic discontinuity ina continuously distributed species: mitochondrial DNA in the American oyster, *Crussostrea virginica*. Genetics, 124, 397–406.

Richardson, B., Hayes, R., Wheeler, S., and Yardin, M. 2002. Social structures, genetic structures and dispersal strategies in Australian rabbit (*Oryctolagus cuniculus*) populations. Behavioral Ecology and Sociobiology, 51(2), 113–121.

Riginos, C., and Henzler, C.M. 2008. Patterns of mtDNA diversity in North Atlantic populations of the mussel *Mytilus edulis.* Marine Biology, 155(4), 399–412.

Rising, J.D., and Avise, J.C. 1993. Application of genealogical-concordance principles to the taxonomy and evolutionary history of the sharp-tailed sparrow (*Ammodramus caudacutus*). The Auk, 110(4), 844–856.

Rohwer, S., and Wood, C. 1998. Three hybrid zones between hermit and Townsend’s warblers in Washington and Oregon. The Auk, 115(2), 284–310.

Rose, C.G., Paynter, K.T., and Hare, M.P. 2006. Isolation by distance in the eastern oyster, *Crassostrea virginica*, in Chesapeake Bay. Journal of Heredity, 97(2), 158–170.

Ruegg, K. 2008. Genetic, morphological, and ecological characterization of a hybrid zone that spans a migratory divide. Evolution, 62(2), 452–466.

Ruegg, K.C., and Smith, T.B. 2002. Not as the crow flies: a historical explanation for circuitous migration in Swainson’s thrush (*Catharus ustulatus*). Proceedings of the Royal Society of London. Series B: Biological Sciences, 269(1498), 1375–1381

Rush, A.C., Cannings, R.J., and Irwin, D.E. 2009. Analysis of multilocus DNA reveals hybridization in a contact zone between *Empidonax* flycatchers. Journal of Avian Biology, 40(6), 614–624.

Ryan, S.F., Fontaine, M.C., Scriber, J.M., Pfrender, M.E., O’Neil, S.T., and Hellmann, J.J. 2017. Patterns of divergence across the geographic and genomic landscape of a butterfly hybrid zone associated with a climatic gradient. Molecular Ecology, 26(18), 4725–4742.

Santucci, F., Nascetti, G., and Bullini, L. 1996. Hybrid zones between two genetically differentiated forms of the pond frog *Rana lessonae* in southern Italy. Journal of Evolutionary Biology, 9(4), 429–450.

Schultze, N., Laufer, H., Kindler, C., and Fritz, U. 2019. Distribution and hybridisation of barred and common grass snakes (*Natrix helvetica, N. natrix*) in Baden-Württemberg, South-western Germany. Herpetozoa, 32, 229–236.

Schultze, N., Spitzweg, C., Corti, C., Delaugerre, M., Di Nicola, M.R., Geniez, P., Lapini, L., Liuzzi, C., Lunghi, E., Novarini, N., Picariello, O., Razzetti, E., Sperone, E., Stellati, L., Vignoli, L., Asztalos, M., Kindler, C., Vamberger, M., and Fritz, U. 2020. Mitochondrial ghost lineages blur phylogeography and taxonomy of *Natrix helvetica* and *N. natrix* in Italy and Corsica. Zoologica Scripta, 49(4), 395–411.

Seabrook-Davison, M., Huynen, L., Lambert, D.M., and Brunton, D.H. 2009. Ancient dna resolves identity and phylogeny of new zealand’s extinct and living quail (*Coturnix* sp.). PLoS ONE, 4(7), e6400.

Seneviratne, S.S., Toews, D.P.L., Brelsford, A., and Irwin, D.E. 2012. Concordance of genetic and phenotypic characters across a sapsucker hybrid zone. Journal of Avian Biology, 43(2), 119–130.

Sequeira, F., Alexandrino, J., Rocha, S., Arntzen, J.W., and Ferrand, N. 2005. Genetic exchange across a hybrid zone within the Iberian endemic golden-striped salamander, *Chioglossa lusitanica.* Molecular Ecology, 14(1), 245–254.

Sequeira, F., Arntzen, J.W., van Gulik, D., Hajema, S., Diaz, R.L., Wagt, M., and van Riemsdijk, I. 2022. Genetic traces of hybrid zone movement across a fragmented habitat. Journal of Evolutionary Biology, 35(3), 400–412.

Seyoum, S., Gandy, R.L., Crowley, C.E., and Puchulutegui, C. 2021. A novel interpretation of speciation, hybridization, and genetic population structure of the stone crabs *Menippe mercenaria* (Say, 1817–1818) and M. adina Williams & Felder, 1986 (Decapoda: Brachyura: Menippidae). Journal of Crustacean Biology, 41(2), ruab018.

Singhal, S., and Moritz, C. 2013. Reproductive isolation between phylogeographic lineages scales with divergence. Proceedings of the Royal Society B: Biological Sciences, 280(1772), 20132246.

Slager, D.L., Epperly, K.L., Ha, R.R., Rohwer, S., Wood, C., Hemert, C., and Klicka, J. 2020. Cryptic and extensive hybridization between ancient lineages of American crows. Molecular Ecology, 29(5), 956–969.

Smith, M.A., and M. Green, D. 2005. Dispersal and the metapopulation paradigm in amphibian ecology and conservation: are all amphibian populations metapopulations? Ecography, 28(1), 110–128.

Stewart, K.A., Austin, J.D., Zamudio, K.R., and Lougheed, S.C. 2016. Contact zone dynamics during early stages of speciation in a chorus frog (*Pseudacris crucifer*). Heredity, 116(2), 239–247.

Stuckas, H., Stoof, K., Quesada, H., and Tiedemann, R. 2009. Evolutionary implications of discordant clines across the Baltic Mytilus hybrid zone (*Mytilus edulis* and *Mytilus trossulus*). Heredity, 103(2), 146–156.

Sun, C. 1997. Dispersal of young in red squirrels (*Tamiasciurus hudsonicus*). American Midland Naturalist, 138(2), 252.

Sutter, A., Beysard, M., and Heckel, G. 2013. Sex-specific clines support incipient speciation in a common European mammal. Heredity, 110(4), 398–404.

Suzuki, H., Nunome, M., Kinoshita, G., Aplin, K.P., Vogel, P., Kryukov, A.P., Jin, M.-L., Han, S.-H., Maryanto, I., Tsuchiya, K., Ikeda, H., Shiroishi, T., Yonekawa, H., and Moriwaki, K. 2013. Evolutionary and dispersal history of Eurasian house mice *Mus musculus* clarified by more extensive geographic sampling of mitochondrial DNA. Heredity, 111(5), 375–390.

Szymura, J.M., and Barton, N.H. 1986. Genetic analysis of a hybrid zone between the fire‐bellied toads, *Bombina bombina* and *B. variegata*, near Cracow in southern Poland. Evolution, 40(6), 1141–1159.

Tarnowska, E., Niedziałkowska, M., Gerc, J., Korbut, Z., Górny, M., and Jędrzejewska, B. 2016. Spatial distribution of the Carpathian and eastern mtDNA lineages of the bank vole in their contact zone relates to environmental conditions. Biological Journal of the Linnean Society, 119(3), 732–744.

Tarwater, C.E. 2012. Influence of phenotypic and social traits on dispersal in a family living, tropical bird. Behavioral Ecology, 23(6), 1242–1249.

Taylor, S.A., Anderson, D.J., Zavalaga And, C.B., and Friesen, V.L. 2012. Evidence for strong assortative mating, limited gene flow, and strong differentiation across the blue‐footed/Peruvian booby hybrid zone in northern Peru. Journal of Avian Biology, 43(4), 311–324.

Teeter, K.C., Payseur, B.A., Harris, L.W., Bakewell, M.A., Thibodeau, L.M., O’Brien, J.E., Krenz, J.G., Sans-Fuentes, M.A., Nachman, M.W., and Tucker, P.K. 2008. Genome-wide patterns of gene flow across a house mouse hybrid zone. Genome Research, 18(1), 67–76.

Teeter, K.C., Thibodeau, L.M., Gompert, Z., Buerkle, C.A., Nachman, M.W., and Tucker, P.K. 2010. The variable genomic architecture of isolation between hybridizing species of house mice. Evolution, 64(2), 472–485.

Thorpe, R.S., Surget-Groba, Y., and Johansson, H. 2008. The relative importance of ecology and geographic isolation for speciation in anoles. Philosophical Transactions of the Royal Society B: Biological Sciences, 363(1506), 3071–3081.

Toews, D.P.L., Brelsford, A., and Irwin, D.E. 2011. Hybridization between Townsend’s *Dendroica townsendi* and black-throated green warblers *D. virens* in an avian suture zone. Journal of Avian Biology, 42(5), 434–446.

Toews, D.P.L., Mandic, M., Richards, J.G., and Irwin, D.E. 2014. Migration, mitochondria, and the yellow-rumped warbler. Evolution, 68(1), 241–255.

Tominaga, A., Matsui, M., and Matsui, M. 2021. Structure and movement of the hybrid zone between two divergent lineages of the Japanese newt *Cynops pyrrhogaster* (Amphibia: Urodela) in Central Japan. Journal of Zoological Systematics and Evolutionary Research, 59(5), 1097–1112.

Tominaga, A., Matsui, M., Yoshikawa, N., Eto, K., and Nishikawa, K. 2018. Genomic displacement and shift of the hybrid zone in the Japanese fire-bellied newt. Journal of Heredity, 109(3), 232–242.

Tominaga, A., Matsui, M., Yoshikawa, N., Nishikawa, K., Hayashi, T., Misawa, Y., Tanabe, S., and Ota, H. 2013. Phylogeny and historical demography of *Cynops pyrrhogaster* (Amphibia: Urodela): Taxonomic relationships and distributional changes associated with climatic oscillations. Molecular Phylogenetics and Evolution, 66(3), 654–667.

van Riemsdijk, I., Butlin, R.K., Wielstra, B., and Arntzen, J.W. 2019. Testing an hypothesis of hybrid zone movement for toads in France. Molecular Ecology, 28(5), 1070–1083.

Vazquez, P., Cooper, S.J.B., Gosalvez, J., and Hewitt, G.M. 1994. Nuclear DNA introgression across a Pyrenean hybrid zone between parapatric subspecies of the grasshopper *Chorthippus parallelus*. Heredity, 73(4), 436–443.

Vercken, E., Sinervo, B., and Clobert, J. 2012. The importance of a good neighborhood: dispersal decisions in juvenile common lizards are based on social environment. Behavioral Ecology, 23(5), 1059–1067.

Virdee, S.R., and Hewitt, G.M. 1994. Clines for hybrid dysfunction in a grasshopper hybrid zone. Evolution, 48(2), 392–407.

Wahlberg, N. 2006. That awkward age for butterflies: insights from the age of the butterfly subfamily Nymphalinae (Lepidoptera: Nymphalidae). Systematic Biology, 55(5), 703–714.

Walsh, J., Billerman, S.M., Rohwer, V.G., Butcher, B.G., and Lovette, I.J. 2020. Genomic and plumage variation across the controversial Baltimore and Bullock’s oriole hybrid zone. The Auk, 137(4), ukaa044.

Walsh, J., Kovach, A.I., Babbitt, K.J., and O’Brien, K.M. 2012. Fine-scale population structure and asymmetrical dispersal in an obligate salt-marsh passerine, the saltmarsh sparrow (*Ammodramus caudacutus*). The Auk, 129(2), 247–258.

Walsh, J., Shriver, W.G., Correll, M.D., Olsen, B.J., Elphick, C.S., Hodgman, T.P., Rowe, R.J., O’Brien, K.M., and Kovach, A.I. 2017. Temporal shifts in the saltmarsh–Nelson’s sparrow hybrid zone revealed by replicated demographic and genetic surveys. Conservation Genetics, 18(2), 453–466.

Walsh, J., Shriver, W.G., Olsen, B.J., and Kovach, A.I. 2016. Differential introgression and the maintenance of species boundaries in an advanced generation avian hybrid zone. BMC Evolutionary Biology, 16(1), 65.

Wang, L., Luzynski, K., Pool, J.E., Janoušek, V., Dufková, P., Vyskočilová, M.M., Teeter, K.C., Nachman, M.W., Munclinger, P., Macholán, M., Piálek, J., and Tucker, P.K. 2011. Measures of linkage disequilibrium among neighbouring SNPs indicate asymmetries across the house mouse hybrid zone. Molecular Ecology, 20(14), 2985–3000.

Weir, J.T., and Schluter, D. 2004. Ice sheets promote speciation in boreal birds. Proceedings of the Royal Society of London. Series B: Biological Sciences, 271(1551), 1881–1887.

Weir, J.T., Faccio, M.S., Pulido-Santacruz, P., Barrera-Guzmán, A.O., and Aleixo, A. 2015. Hybridization in headwater regions, and the role of rivers as drivers of speciation in Amazonian birds. Evolution, 69(7), 1823–1834.

Wen, G., and Fu, J. 2021. Isolation and reconnection: demographic history and multiple contact zones of the green odorous frog (*Odorrana margaretae*) around the Sichuan Basin. Molecular Ecology, 30(16), 4103–4117.

While, G.M., Michaelides, S., Heathcote, R.J.P., MacGregor, H.E.A., Zajac, N., Beninde, J., Carazo, P., Pérez I De Lanuza, G., Sacchi, R., Zuffi, M.A.L., Horváthová, T., Fresnillo, B., Schulte, U., Veith, M., Hochkirch, A., and Uller, T. 2015. Sexual selection drives asymmetric introgression in wall lizards. Ecology Letters, 18(12), 1366–1375.

Wielstra, B., Burke, T., Butlin, R.K., and Arntzen, J.W. 2017a. A signature of dynamic biogeography: enclaves indicate past species replacement. Proceedings of the Royal Society B: Biological Sciences, 284(1868), 20172014.

Wielstra, B., Burke, T., Butlin, R.K., Avcı, A., Üzüm, N., Bozkurt, E., Olgun, K., and Arntzen, J.W. 2017b. A genomic footprint of hybrid zone movement in crested newts. Evolution Letters, 1(2), 93–101.

Wolfgramm, H., Martens, J., Töpfer, T., Vamberger, M., Pathak, A., Stuckas, H., and Päckert, M. 2021. Asymmetric allelic introgression across a hybrid zone of the coal tit (*Periparus ater*) in the central Himalayas. Ecology and Evolution, 11(23), 17332–17351.

Yanchukov, A., Hofman, S., Szymura, J.M., Mezhzherin, S.V., Morozov‐Leonov, S.Y., Barton, N.H., and Nürnberger, B. 2006. Hybridization of *Bombina bombina* and *B. variegata* (Anura, Discoglossidae) at a sharp ecotone in western Ukraine: comparisons across transects and over time. Evolution, 60(3), 583–600.

Yang, W., Feiner, N., Laakkonen, H., Sacchi, R., Zuffi, M.A.L., Scali, S., While, G.M., and Uller, T. 2020. Spatial variation in gene flow across a hybrid zone reveals causes of reproductive isolation and asymmetric introgression in wall lizards. Evolution, 74(7), 1289–1300.

Yannic, G., Basset, P., and Hausser, J. 2008. A hybrid zone with coincident clines for autosomal and sex‐specific markers in the *Sorex araneus* group. Journal of Evolutionary Biology, 21(3), 658–667.

Yannic, G., Basset, P., and Hausser, J. 2009. Chromosomal rearrangements and gene flow over time in an inter-specific hybrid zone of the *Sorex araneus* group. Heredity, 102(6), 616–625.

Zann, R., and Runciman, D. 1994. Survivorship, dispersal and sex ratios of zebra finches *Taeniopygia guttata* in southeast Australia. Ibis, 136(2), 136–143.

Zhou, W., Wen, Y., Fu, J., Xu, Y., Jin, J., Ding, L., Min, M., Che, J., and Zhang, Y. 2012. Speciation in the *Rana chensinensis* species complex and its relationship to the uplift of the Qinghai–Tibetan Plateau. Molecular Ecology, 21(4), 960–973.

Zieliński, P., Dudek, K., Arntzen, J.W., Palomar, G., Niedzicka, M., Fijarczyk, A., Liana, M., Cogǎlniceanu, D., and Babik, W. 2019. Differential introgression across newt hybrid zones: evidence from replicated transects. Molecular Ecology, 28(21), 4811–4824.

Zieliński, P., Nadachowska-Brzyska, K., Wielstra, B., Szkotak, R., Covaciu-Marcov, S.D., Cogălniceanu, D., and Babik, W. 2013. No evidence for nuclear introgression despite complete mtDNA replacement in the Carpathian newt (*Lissotriton montandoni*). Molecular Ecology, 22(7), 1884–1903.
